# Chemogenetic profiling reveals PP2A‐independent cytotoxicity of proposed PP2A activators iHAP1 and DT‐061

**DOI:** 10.15252/embj.2022110611

**Published:** 2022-06-13

**Authors:** Gianmatteo Vit, Joana Duro, Girish Rajendraprasad, Emil P T Hertz, Lya Katrine Kauffeldt Holland, Melanie Bianca Weisser, Brennan C McEwan, Blanca Lopez‐Mendez, Paula Sotelo‐Parrilla, A Arockia Jeyaprakash, Guillermo Montoya, Niels Mailand, Kenji Maeda, Arminja Kettenbach, Marin Barisic, Jakob Nilsson

**Affiliations:** ^1^ Novo Nordisk Foundation Center for Protein Research Faculty of Health and Medical Sciences University of Copenhagen Copenhagen Denmark; ^2^ Cell Division and Cytoskeleton Danish Cancer Society Research Center Copenhagen Denmark; ^3^ Cell Death and Metabolism Unit Center for Autophagy, Recycling and Disease (CARD) Danish Cancer Society Research Center (DCRC) Copenhagen Denmark; ^4^ Department of Biochemistry and Cell Biology Geisel School of Medicine at Dartmouth College Hanover NH USA; ^5^ Norris Cotton Cancer Center Lebanon NH USA; ^6^ Wellcome Trust Center for Cell Biology University of Edinburgh Edinburgh UK; ^7^ Department of Cellular and Molecular Medicine Faculty of Health Sciences University of Copenhagen Copenhagen Denmark

**Keywords:** chemogenic profiling, DT‐061, iHAP1, phosphatase, PP2A, Pharmacology & Drug Discovery, Post-translational Modifications & Proteolysis

## Abstract

Protein phosphatase 2A (PP2A) is an abundant phosphoprotein phosphatase that acts as a tumor suppressor. For this reason, compounds able to activate PP2A are attractive anticancer agents. The compounds iHAP1 and DT‐061 have recently been reported to selectively stabilize specific PP2A‐B56 complexes to mediate cell killing. We were unable to detect direct effects of iHAP1 and DT‐061 on PP2A‐B56 activity in biochemical assays and composition of holoenzymes. Therefore, we undertook genome‐wide CRISPR‐Cas9 synthetic lethality screens to uncover biological pathways affected by these compounds. We found that knockout of mitotic regulators is synthetic lethal with iHAP1 while knockout of endoplasmic reticulum (ER) and Golgi components is synthetic lethal with DT‐061. Indeed we showed that iHAP1 directly blocks microtubule assembly both *in vitro* and *in vivo* and thus acts as a microtubule poison. In contrast, DT‐061 disrupts both the Golgi apparatus and the ER and lipid synthesis associated with these structures. Our work provides insight into the biological pathways perturbed by iHAP1 and DT‐061 causing cellular toxicity and argues that these compounds cannot be used for dissecting PP2A‐B56 biology.

## Graphical abstract

Cellular toxicity of two small‐molecule compounds is based on microtubule depolymerization and Golgi/ER disruption, respectively, rather than protein phosphatase 2A activation.

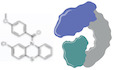

## Introduction

Protein phosphatase 2A (PP2A) is an abundant phosphoprotein phosphatase (PPP) and accounts for much of the serine/threonine phosphatase activity in eukaryotic cells, thereby suppressing multiple oncogenic kinase pathways (Westermarck & Hahn, 2008; Kauko & Westermarck, 2018; Westermarck, 2018; Vainonen *et al*, 2021). The PP2A holoenzyme consists of a catalytic subunit (PP2AC, A and B isoforms), a scaffold subunit (PPP2R1, A and B isoforms) and one of four regulatory B subunits (B55α‐δ, B56α‐ε, PR72/PR130 or STRN1‐4, multiple isoforms of each B subunit) (Virshup, 2000; Hertz *et al*, 2016; Nilsson, 2019). The B subunits confer specificity to PP2A holoenzymes by directly binding to substrates or substrate specifiers (Cundell *et al*, 2016; Hertz *et al*, 2016). Given that PP2A activity is suppressed in cancers either through overexpression of inhibitory proteins or through mutations in PP2A components, there is an interest in developing compounds that can reactivate PP2A for cancer treatment (Westermarck & Neel, 2020; Vainonen *et al*, 2021).

**Figure 1:**
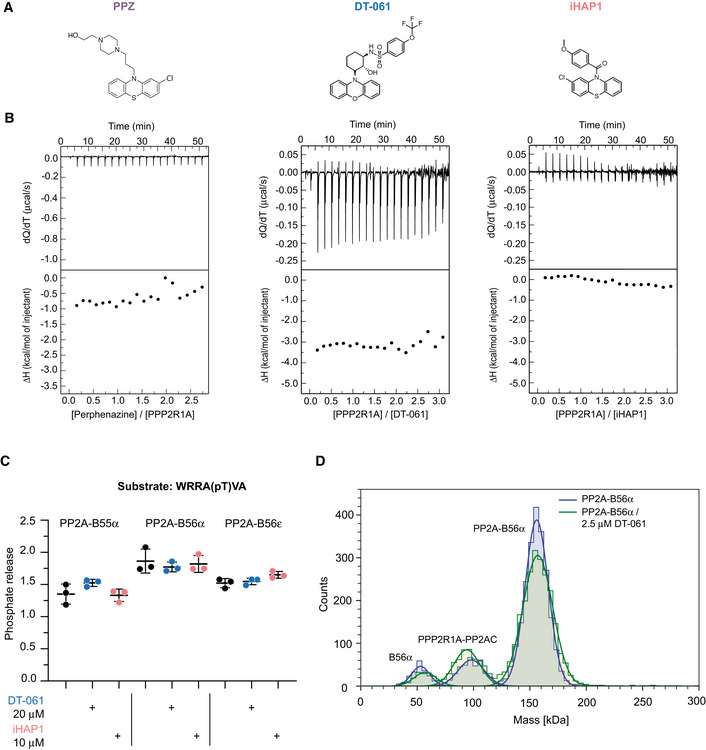
Perphenazine, DT‐061 and iHAP1 have no direct effect on PP2A holoenzymes Chemical structure of perphenazine, DT‐061 and iHAP1.Thermogram (top panel) for the calorimetric titration of perphenazine (400 μM, in syringe) in PPP2R1A (30 μM, in calorimetric cell) or PPP2R1A (300 μM, in syringe) in DT‐061 (20 μM, in calorimetric cell) or iHAP1 (20 μM, in calorimetric cell) at 25°C and corresponding ligand‐normalized integrated heats (bottom panel). Experiments were performed in 5% DMSO. At least two independent experiments were performed. Control injection of PPP2R1A shown in Fig EV1F.
*In vitro* dephosphorylation assay using a basofilic phosphopeptide and purified PP2A holoenzymes. The peptide was incubated with specific PP2A holoenzymes together with iHAP1 or DT‐061 as indicated. The reaction was stopped after 15 min, and the amount of phosphate released directly measured. Mean and SD from three technical replicates of one out of three independent experiments are shown.MP mass distribution of the PP2A‐B56α holoenzyme at a final concentration of 50 nM in absence (blue) and in presence (green) of 2.5 µM DT‐061 in PBS. The mass photometry measurement of the holoenzyme shows a similar distribution of species in presence or absence of DT‐061. A representative experiment of two independent experiments is shown.

Several compounds based on a phenothiazine or phenoxazine scaffold, which are both tricyclic heterocycle structures and include perphenazine (PPZ), iHAP1 and DT‐061, have been shown to modulate PP2A holoenzyme stoichiometry/activity, thus mediating their anticancer activity (Fig 1A) (Gutierrez *et al*, 2014; Leonard *et al*, 2020; Morita *et al*, 2020). In a screen for FDA‐approved compounds that kill T‐cell acute lymphoblastic leukemia (T‐ALL) cells, the antipsychotic drug perphenazine could be identified (Gutierrez *et al*, 2014). Through mass spectrometry‐based approaches, the target of perphenazine was argued to be PPP2R1A. In a parallel work, tricyclic heterocyclic compounds that affected FoxO1 localization were identified and reengineered to remove their effects on the central nervous system, making them more cancer specific (Kastrinsky *et al*, 2015). This reengineering led to the identification of several compounds (small‐molecule activators of PP2A, SMAPs), including DT‐061, that were found to bind PPP2R1A with a Kd of 235 nM, and the binding site mapped to amino acids 194‐198 of PPP2R1A. In *in vitro* assays, 20 μM DT‐061 was found to bind and activate PP2A‐B56γ and the PPP2R1A‐PP2AC complex by 20–30% (Sangodkar *et al*, 2017).

In two recent publications, perphenazine and DT‐061 discoveries were extended (Leonard *et al*, 2020; Morita *et al*, 2020). Morita *et al* (2020) improved the perphenazine scaffold to remove, among others, off‐target effects on microtubules to generate a novel compound named iHAP1 (Morita *et al*, 2020). The identification of iHAP1 was in part based on the screening of a compound library by adding compounds to cells for 3 h, followed by purification of affinity‐tagged PP2AC coupled with activity testing. It was found that iHAP1 specifically stabilizes PP2A‐B56ε in leukemic and Kelly cells and that this causes a strong arrest in prometaphase through the selective dephosphorylation of MYBL2 by PP2A‐B56ε. DT‐061 mechanism of action was also investigated by Morita *et al* (2020) using a similar approach, and they found that the target is PP2A‐B55α (Morita *et al*, 2020). These results contrast with Leonard *et al* (2020) that carried out a structure‐function analysis of DT‐061 and identified PP2A‐B56α as the target of DT‐061 (Leonard *et al*, 2020). A PP2A‐B56α‐DT‐061 complex was analyzed by cryo‐EM, and the analysis revealed that DT‐061 interacts with an interface formed by all the three subunits of the PP2A‐B56α holoenzyme. This interface is close to the very C‐terminal flexible tail of the PP2AC subunit, and specific residues in B56α allow for accommodation of DT‐061, explaining the selective binding to PP2A‐B56α and not to the other B56 isoforms.

Here, we extensively characterized iHAP1 and DT‐061 and explored the mechanisms of cellular toxicity using chemogenic profiling. Our results argue that these compounds mediate cellular toxicity independently of targeting PP2A‐B56 complexes.

## Results

### Tricyclic heterocyclic compounds do not bind PPP2R1A

**Figure EV1:**
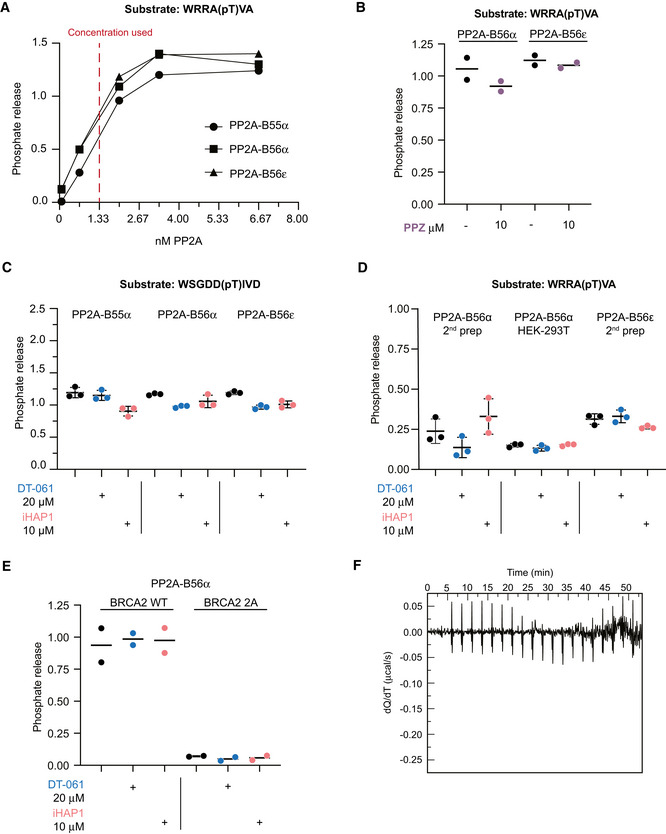
Perphenazine, iHAP1 and DT‐061 do not activate PP2A complexes *in vitro* ATitration experiment of different PP2A holoenzyme preparations to determine appropriate concentration to use in enzymatic assays. Experiment was performed twice using the concentrations of enzyme stated in the figure. Shown is one representative experiment.B–E
*In vitro* dephosphorylation assays using model phosphopeptides and purified PP2A holoenzymes. The phosphopeptides were incubated with specific PP2A holoenzymes together with PPZ, iHAP1 or DT‐061 at the indicated concentrations. The reaction was stopped after 15 min, and the amount of phosphate released was measured. In (E) substrates are BRCA2 WT: P(pS)QKAEITELSTILEESGSQW and BRCA2 2A: P(pS)QKAEITEASTALEESGSQW. Mean and SD from three technical replicates of one out of three independent experiments is shown. Experiment in (E) is representative of two independent experiments.FInjection of PPP2R1A (300 μM) into the ITC buffer (5% DMSO) used for the DT‐061, iHAP experiments. The experiments were performed at 25°C. No significant effects due to dilution of protein (control experiment for Fig 1B)

We initially characterized perphenazine which was the first compound reported to act on PP2A (Gutierrez *et al*, 2014). As the original characterization of perphenazine as a PP2A activator contained no experimental data confirming direct binding, we found this important to investigate. By isothermal titration calorimetry (ITC) and NMR, we were unable to detect any specific interaction between PPP2R1A and perphenazine, and we observed no effect on PP2A‐B56α/ε activity in *in vitro* reconstituted phosphatase assays (Figs 1B and EV1A and B, and Appendix Figs S1–S3). Based on this result, we decided to further investigate iHAP1 and DT‐061 using similar approaches. We did not detect any effect of iHAP1 and DT‐061 on PP2A holoenzyme activity *in vitro* and failed to reproduce specific binding of these compounds to PPP2R1A by ITC and NMR (Figs 1B and C, and EV1C–F and Appendix Figs S1, S2, S4 and S5). We next investigated if DT‐061 has a stabilizing effect on purified PP2A‐B56α holoenzyme using mass photometry which can quantitatively measure composition of protein solutions. We observed some dissociation of purified PP2A‐B56α at a concentration of 50 nM, but this dissociation was not prevented by the addition of DT‐061 (Fig 1D).

Collectively, we were unable to confirm direct effects of tricyclic heterocyclic compounds on PP2A complexes.

### The cellular toxicity of iHAP1 and DT‐061 is not dependent on specific B56 subunits

**Figure 2:**
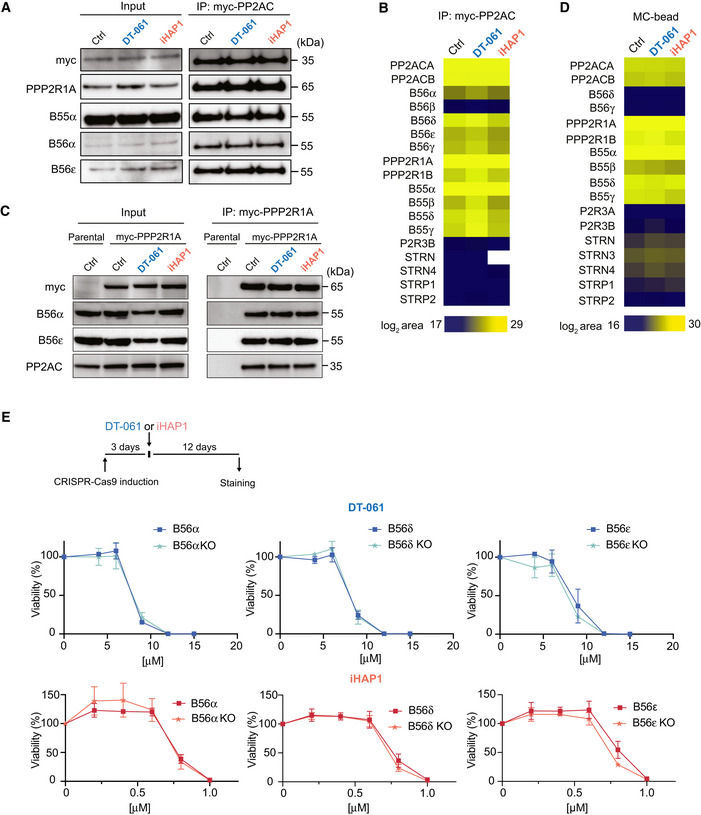
DT‐061 and iHAP1 toxicity is not affected by knockdown of specific B56 subunits A–CA stable inducible HEK‐293T cell line expressing myc‐PP2AC (panel A and B) and a stable inducible HeLa cell line expressing myc‐PP2R1A (C) were treated with DMSO, 20 μM DT‐061 or 2 μM iHAP1 for 30 min or 2 h respectively and myc‐PP2AC and myc‐PP2R1A affinity purified, respectively. The binding of the indicated proteins was analyzed by Western blotting (A and C) and myc‐PP2AC by quantitative mass spectrometry (B). (A) Representative of three independent experiments and (C) representative of two technical repeats and (B) analysis of a biological triplicate.DHEK‐293T cells were treated with DMSO, 20 μM DT‐061 or 2 μM iHAP1 for 2 h, and protein lysates were incubated with a microcystin affinity column to capture PPP complexes. Bound complexes were analyzed by mass spectrometry to identify PP2A components. Analysis conducted on a biological triplicate.EDoxycyclin‐inducible CRISPR‐Cas9 HeLa cell lines allow for depletion of specific B56 subunits. Growth assays in the presence of the indicated concentrations of DT‐061 or iHAP1 measured after 12 days of treatment. Mean and SD of three independent experiments are shown. Source data are available online for this figure.

Given that we could not confirm an effect of the compounds on PP2A activity *in vitro*, we set out to investigate the impact on PP2A holoenzyme formation in cells. Incubation with iHAP1 or DT‐061 has been shown to affect PP2A holoenzyme stoichiometries in cells through selective stabilization of PP2A‐B56ε and PP2A‐B56α, respectively (Leonard *et al*, 2020; Morita *et al*, 2020). We therefore treated HEK‐293T cells stably expressing myc‐tagged PP2AC with 2 μM iHAP1 or 20 μM DT‐061 for 30 min, a timeframe sufficient to induce clear cellular effects (see below), and immunopurified myc‐PP2AC. We have recently shown that myc‐PP2AC is functional as it can complement the RNAi‐mediated depletion of PP2AC (Nasa *et al*, 2020). We did not detect any effect on the binding of tested B subunits with PP2AC by Western blotting or B56 subunits detected by quantitative mass spectrometry analysis (Fig 2A and B, Dataset EV1). We performed a similar experiment using myc‐tagged PPP2AR1, but Western blot analysis for B56α and B56ε did not reveal any changes in holoenzyme composition (Fig 2C). In a complementary approach, we incubated lysates from untreated or iHAP1/DT‐061‐treated HEK‐293T cells with microcystin coupled to beads and analyzed by mass spectrometry the composition of endogenous PPP components which were pulled down by microcystin (Lyons *et al*, 2018) (Fig 2D, Dataset EV2). If a certain PP2A holoenzyme is stabilized by the compound, one would detect this in the unbiased MS interactome analysis. Again, we observed no statistically significant changes in the detection of B56 or B55 subunits (*P*‐value < 0.05, log2 ratio > 1). To further explore the effect of DT‐061 on endogenous PP2A‐B56α, we used size exclusion chromatography of total cell extracts from HeLa or H358 cells. Firstly, these experiments revealed that very little free B56α is present in cells as all B56α fully co‐migrated with PPP2R1A in a ~ 300 kDa complex despite a 5‐fold dilution of total protein concentrations. Secondly, we did not see an increase in the ratio of B56α/PPP2R1A in the peak fractions upon addition of DT‐061 to cells for 2 h (Appendix Figs S6 and S7).

**Figure EV2:**
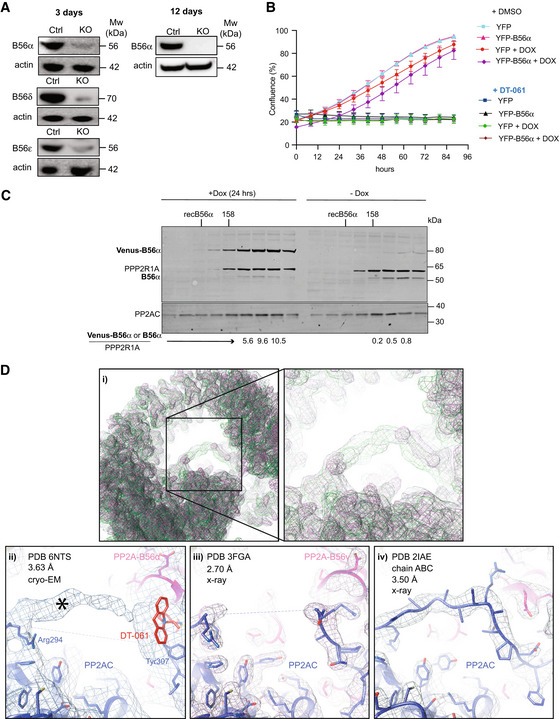
*In vivo* and structural analysis of drug activity on cells CRISPR‐Cas9‐inducible HeLa cell lines for PP2A regulatory subunits. Western blotting shows the removal of the indicated B56 subunits 3 and 12 days after addition of doxycycline, respectively. Actin was used as loading control. At least three biological replicates of the experiment were performed.Incucyte growth assay using a HeLa cell line stably expressing inducible YFP‐B56α. Induced cells were treated with Doxycycline for 12 h before imaging. Cell was treated with 20 µM DT‐061 or DMSO (Ctrl). Experiment was repeated in three times independently of each other with each experiment performed in technical triplicate. A representative independent experiment is shown with mean and SD.A total cell extract was prepared from HeLa cells stably expressing doxycycline‐inducible YFP‐B56α. Cells were either untreated or expression induced for 24 h with Doxycycline and the complexes in the cell extracts separated on a Superdex200 column. The migration of molecular weight markers or recombinant untagged B56α is indicated on top. The fractions were analyzed by quantitative Licor western blot for PP2A/A, B56α and PP2A/C. The ratio of B56α or Venus‐B56α to PP2A/A in the peak fractions is shown below. PP2AC levels co‐migrating with PPP2R1A increased 2–3‐fold in the sample where YFP‐B56α was induced. Experiment was performed once.(i) The superposition of the crystallographic electron densities maps of PDB 2IAE chain a,b,c (green) and PDB 3FGA (light pink) with cryo‐EM map EMD‐0510 (gray) at a map threshold of 0.25 shows that in all three maps, density is protruding into the center of the horseshoe‐shaped PP2A holoenzyme at the same position (closeup, right). The fits of all three PP2A structures—PDB 6NTS (ii), PDB 3FGA (iii) and PDB 2IAE, (chain a,b,c) (iv) —into their respective densities show that in all three cases, this area of density was attributed to the C‐terminal tail of the PP2A catalytic subunit. In the case of PDB 6NTS, the overall map quality and local resolution in that area reveals less easily interpretable features than in the case of PDBs 3FGA/2IAE. Only the last three C‐terminal tail residues (307‐9) were built, and the DT061 ligand (red) was modeled right next to them. This leaves most of the visible extra density (asterisk) uninterpreted (ii), in a position where in the higher resolution models of PP2A/C (3FGA/2IAE) further residues of the PP2A/C C‐terminus have been placed.

To explore if the cellular toxicity of DT‐061 and iHAP1 is dependent on specific PP2A‐B56 complexes, an inducible CRISPR‐Cas9 system was used (McKinley & Cheeseman, 2017). We induced the expression of gRNAs to B56α, B56δ and B56ε in HeLa cells stably expressing Cas9, which resulted in a large fraction of the cell population becoming full null for these specific B56 subunits for up to 12 days (Fig EV2A). We then monitored the effect of iHAP1 and DT‐061 on cell viability and determined LC50. We observed no effect on LC50 values when the proposed targets of iHAP1 and DT‐061 were removed by CRISPR, arguing that the toxicity of these compounds is unrelated to the stabilization and activation of these specific PP2A‐B56 complexes (Fig 2E). We further tested the hypothesis that increased levels of PP2A‐B56α would result in cellular toxicity using a HeLa cell line where we can induce the expression of YFP‐B56α (Wang *et al*, 2020; Ueki *et al*, 2021). We have previously shown that this YFP‐B56α is functional as it can rescue the cellular phenotypes upon RNAi‐mediated depletion of all B56 isoforms. In this cell line, more than 90% of cells stably express YFP‐B56α upon induction. Indeed, the expression of YFP‐B56α resulted in at least a 10‐fold increase in PP2A‐B56α complexes in cells without affecting cell viability (Fig EV2B and C). Under the same conditions, DT‐061 severely inhibited cell growth.

Based on these results, we revisited the cryo‐EM structure of the proposed PP2A‐B56α‐DT‐061 complex published by Leonard *et al* (2020) (PDB 6NTS) and compared it with previous structural studies of the PP2A‐B56γ complex (PDBs 2NNP (Xu *et al*, 2006), 2IAE (Cho & Xu, 2007) and 3FGA (Xu *et al*, 2009)). This revealed that the assignment of density to DT‐061 is not unambiguous at the given intermediate resolution of 3.6 Å of the cryo‐EM structure (Fig EV2D and Appendix Fig S8). The density assigned to DT‐061 does not fully embed the tricyclic ring of DT‐061, and the compound exhibits steric clashes and low‐quality geometry parameters in Molprobity (Davis *et al*, 2007). In addition, the superposition of PP2A‐B56 structures and their corresponding maps show that the density in that region might as well be ascribed to residues from the adjacent C‐terminal tail of PP2AC, which is known to be flexible and therefore only partially modeled near DT‐061. Therefore, this comparison suggests that the assignment of that density to the ligand is not unambiguous. Structures of unliganded and liganded complexes are needed to fully establish that the assigned density can be attributed to DT‐061 and not residues from the C‐terminal tail as suggested by three previous structures.

Collectively, our analysis indicates that DT‐061 and iHAP1 do not affect specific PP2A‐B56 complexes in cells.

### Genome‐wide CRISPR‐Cas9 synthetic lethality screens identify cellular pathways sensitizing cells to iHAP1 and DT‐061

**Figure 3:**
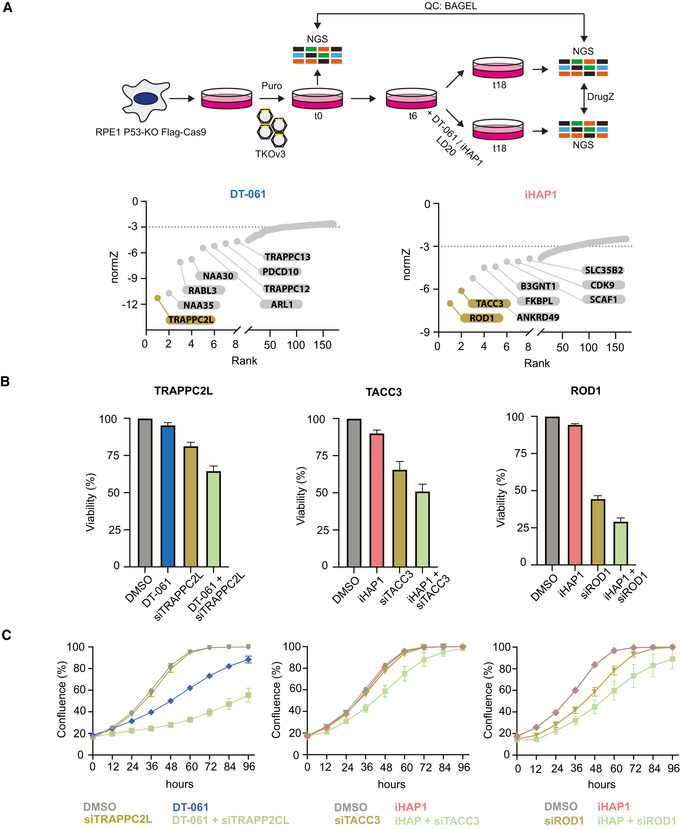
Genome‐wide CRISPR screen establishes sensitizers to DT‐061 and iHAP1 Schematic of the experimental setup and results of CRISPR screen. Genes were ranked based on their normZ score. The top hits which were further analyzed are highlighted.Validation of CRISPR screen results using RNAi depletion followed by SRB growth assay. Cells were treated with DMSO (Ctr) or DT‐061 5.5 µM or iHAP1 0.5 µM.Incucyte growth assay after RNAi depletion of indicated proteins alone or in combination with DMSO or 5.5 μM DT‐061 or 0.5 μM iHAP1 treatment. Data information: (B and C) Mean and SD of three independent experiments are shown. Source data are available online for this figure.

**Figure EV3:**
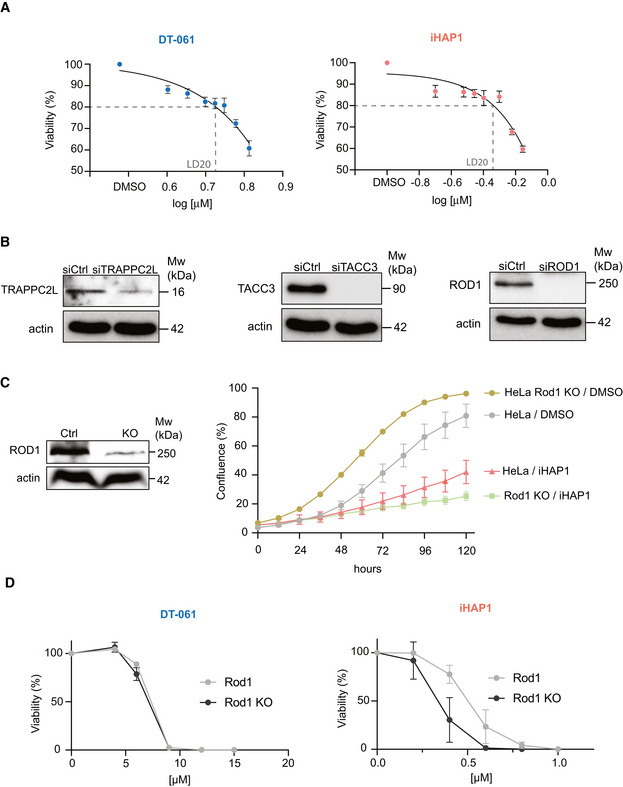
Compound titration and validation of CRISPR screen results Viability of RPE1‐hTERT P53^−/−^ FLAG Cas9 cells with increasing concentrations of iHAP1 or DT‐061 to determine LD20. Mean and SD of three biological replicates is shown.Protein depletion efficiency after RNAi‐treatment. Actin was used as loading control. The experiment was performed three times.Sensitivity of HeLa Rod1 KO cells to 0.5 µM iHAP1. Western blot to confirm Rod1 depletion levels (left) and growth curves for the indicated conditions (right).Sensitivity of HeLa Rod1 KO to DT‐061 or iHAP1 determined using SRB assay. Data information: (C and D) Mean and SD of three independent experiments are shown.

Since iHAP1 and DT‐061 could still be relevant therapeutic compounds for cancer treatment, we set out to identify their mechanisms of cellular toxicity. Genome‐scale CRISPR dropout screens are powerful tools to uncover genetic vulnerabilities to chemical compounds and can provide insight into the molecular pathways affected by these drugs (Shalem *et al*, 2014; Wang *et al*, 2014; Hart *et al*, 2015; Zimmermann *et al*, 2018). To identify genes whose inactivation causes increased sensitivity to iHAP1 or DT‐061, we transduced the RPE1‐hTERT P53^−/−^ Flag‐Cas9 cell line (Olivieri & Durocher, 2021) with the TKOv3 lentiviral library of single‐guide (sg) RNAs targeting 18,053 human genes and monitored the depletion of sgRNAs after 12 days of low‐dose (LD20) drug treatment compared to control treated cells (Figs 3A and EV3A) (Olivieri & Durocher, 2021). A normalized depletion score was calculated for each gene using the DrugZ software (Colic *et al*, 2019). This revealed striking differences in the chemogenetic profile of DT‐061 and iHAP1 (Fig 3A, Dataset EV3). Firstly, we identified no genes related to PP2A biology in our top ranked hits for either of the compounds. Depletion of genes related to Golgi and endoplasmic reticulum (ER) function and integrity, such as components of the NatC complex and TRAPP complex, resulted in increased sensitivity to DT‐061, while known mitotic regulators induced the strongest sensitization to iHAP1 (Fig 3A).

We validated the top hits from our CRISPR screens by an independent method. TRAPPC2L, Rod1 and TACC3 were depleted by RNAi in the RPE1‐hTERT P53^−/−^ Flag‐Cas9 cell line, and sensitivity to iHAP1 and DT‐061 was determined using two different cell growth assays (Figs 3B and C, and EV3B). Consistent with the CRISPR screen, the depletion of TRAPPC2L resulted in increased sensitivity to DT‐061, while depletion of Rod1 and TACC3 resulted in sensitization to iHAP1. We further validated the chemogenic interaction between Rod1 and iHAP1 by monitoring cell growth of a previously generated HeLa Rod1^−/−^ cell line (Zhang *et al*, 2019). As for the RPE1‐hTERT P53^−/−^ background, deletion of Rod1 in HeLa cells caused marked sensitization to iHAP1 but not DT‐061 (Fig EV3C and D).

Collectively, the difference in chemogenic profile between the two drugs argues that the molecular targets of DT‐061 and iHAP1 causing cellular toxicity must be distinct. Based on the chemogenic profiles, we hypothesized that iHAP1 affects mitotic processes while DT‐061 affects Golgi/ER function. We decided to explore this in more detail.

### iHAP1 is a microtubule poison

TACC3 is a microtubule stabilizer during mitosis and an important determinant of the response to microtubule poisons (Schmidt *et al*, 2010; Jost *et al*, 2017). The fact that TACC3 depletion increased the sensitivity to iHAP1 raised the possibility that iHAP1 might affect microtubules directly. Phenothiazine‐derived compounds can disturb microtubule assembly; a previous study had shown that iHAP1 is a microtubule poison (Prinz *et al*, 2011), while Morita *et al* (2020) reported no effect on microtubule polymerization *in vitro*.

**Figure 4:**
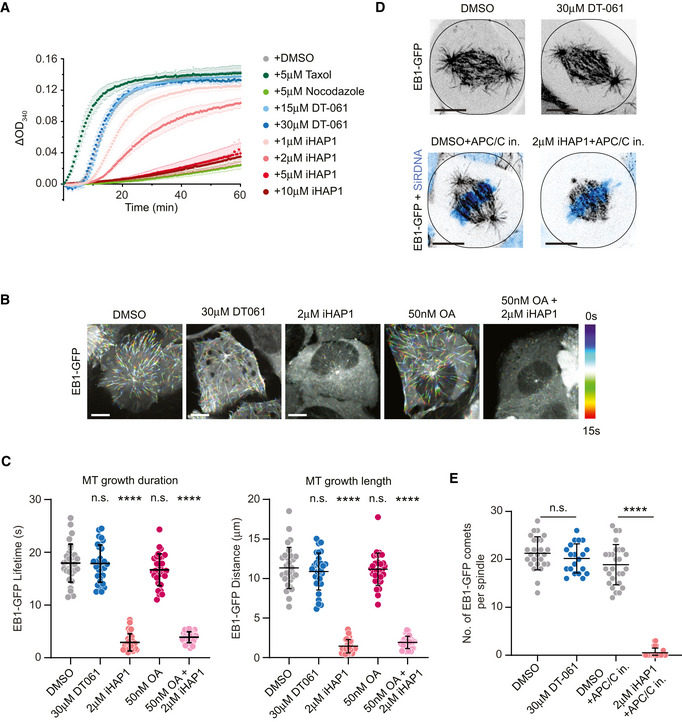
iHAP1 is a microtubule poison *In vitro* tubulin polymerization assay showing concentration dependent effect of iHAP1 on polymerization dynamics. Taxol and Nocodazole served as controls for the experimental setup. The assay was performed in the presence of the indicated drug concentrations. Mean and SEM are shown from three to six independent experiments.Representative color‐coded temporal projection of U2OS EB1‐GFP cells following indicated treatment. Scale bar 10 µm.Quantification of microtubule dynamics acquired from manual tracking of EB1‐GFP comets as indicators for MT growth after drug treatment. The mean and SD are plotted from three independent experiments (*n* = 10 cells per experiment).Representative temporal projection of mitotic spindles of U2OS EB1‐GFP cells following specified treatment. DNA counterstained with SiR‐DNA shown in cyan in merged images. Scale bar 10 µm.Analysis of number of EB1‐GFP comets per mitotic spindle. The mean and SD are plotted from three independent experiments (Total number of cells = 26 (DMSO), 20 (30 µM DT‐061), 27 (DMSO+APC/C in.) and 26 (2 µM iHAP1+APC/C in.)). Data information: *P*‐values were calculated using Student’s *t*‐test or Mann–Whitney *U* test (unpaired, two‐tailed). See Materials and Methods for more details. ns, not significant, ****P < 0.0001. Source data are available online for this figure.

In light of this discrepancy, we tested if iHAP1 has a direct effect on microtubule dynamics. We first conducted *in vitro* microtubule polymerization assays and analyzed the effect of iHAP1 and DT‐061 on this. While DT‐061 had no effect on microtubule polymerization, we observed a clear effect of iHAP1 at 2 μM, the usual concentration in cellular assays. At 5 µM iHAP1, microtubule polymerization was fully blocked, similar to nocodazole treatment, a well‐established microtubule poison (Fig 4A).

**Figure EV4:**
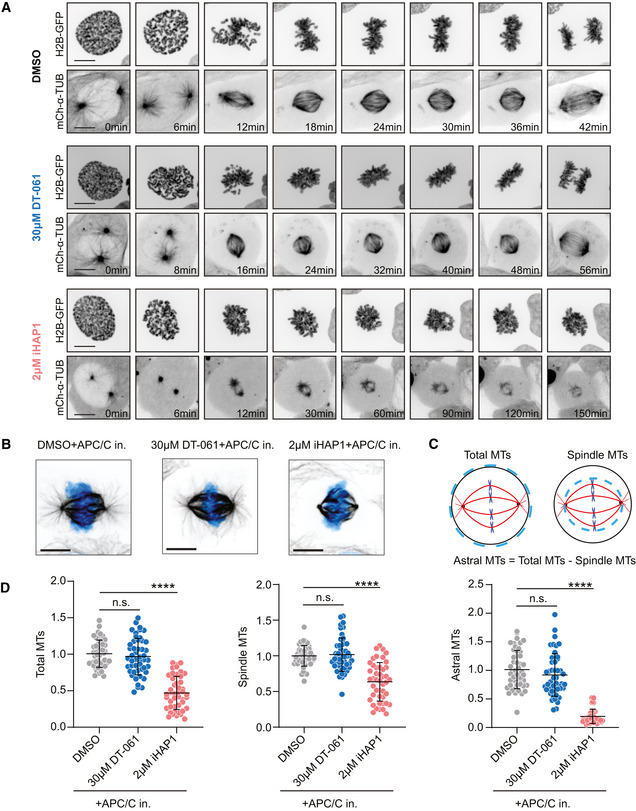
Analysis of mitotic phenotypes in cells treated with DT‐061 or iHAP1 Representative spinning‐disk confocal microscopy time‐series of mitosis in U2OS cells stably expressing H2B‐GFP/mCherry‐α‐tubulin following indicated treatments. Scale bar indicates 10 µm.Representative images of U2OS mitotic spindles in metaphase following indicated treatments, immunostained with α‐tubulin antibody. DNA was counterstained with DAPI (cyan). Scale bar indicates 10 µm.Illustration describing the method used for measuring the amount of total, spindle and astral microtubules.Quantification of the number of microtubules in metaphase‐arrested mitotic cells following indicated treatments. The mean and SD are plotted from three independent experiments (Total no. of cells = 48 (DMSO), 45 (30 µM DT‐061+APC/C in.) and 48 (2 µM iHAP1+APC/C in.)). *P*‐values were calculated using Student’s *t*‐test or Mann–Whitney *U* test (unpaired, two‐tailed). See Materials and Methods for more details. ns, not significant, ****P < 0.0001.

To substantiate our *in vitro* results, we monitored the effect of 2 μM iHAP1 and 30 μM DT‐061 on microtubules in cells. We used a cell line expressing the EB1 microtubule tip tracking protein which allows direct monitoring of *in vivo* microtubule dynamics in a quantitative manner. The addition of iHAP1 to interphase cells for 20 min blocked microtubule growth while DT‐061 had no effect (Fig 4B and C, Movie EV1). The effect of iHAP1 on microtubule growth was not blocked by 50 nM okadaic acid (OA), a potent inhibitor of PP2A activity, which argues that the iHAP1 effect on microtubules is not mediated through effects on PP2A complexes. In agreement with its effect on microtubule dynamics, iHAP1 caused severe chromosome congression defects in mitotic cells shortly upon addition, with consequent spindle collapse and mitotic arrest (Fig EV4A, Movies [Link], [Link], [Link]). To prevent any potential effect of uncongressed chromosomes on analysis of microtubule dynamics in mitotic cells, we arrested cells in metaphase using APC/C inhibitors prior to iHAP1 treatment. Quantification of the number of EB1‐GFP comets within astral microtubules revealed a similar strong effect of iHAP1 on microtubule growth in mitotic cells as well, where it nearly fully abolished astral microtubule polymerization (Figs 4D and E, and EV4B–D). Astral microtubule dynamics in DT‐061‐treated mitotic cells remained unchanged. Moreover, we observed that iHAP1 led to a strong decrease in microtubule intensity on the mitotic spindle and absence of astral microtubules consistent with iHAP1 being a microtubule depolymerizing compound (Fig EV4B–D). Again, this effect on the mitotic spindle was observed 20 min after addition of iHAP1. Consistent with the rest of our data, DT‐061 treatment did not cause any changes in spindle microtubule levels. Collectively, our *in vitro* and *in vivo* characterization uncovers iHAP1 as a microtubule poison, which is consistent with the mitotic arrest the compound induces. The observation that this happens *in vitro* and is nearly instantaneous in cells argues that the effect of iHAP1 is independent of deregulated transcription.

**Figure 5:**
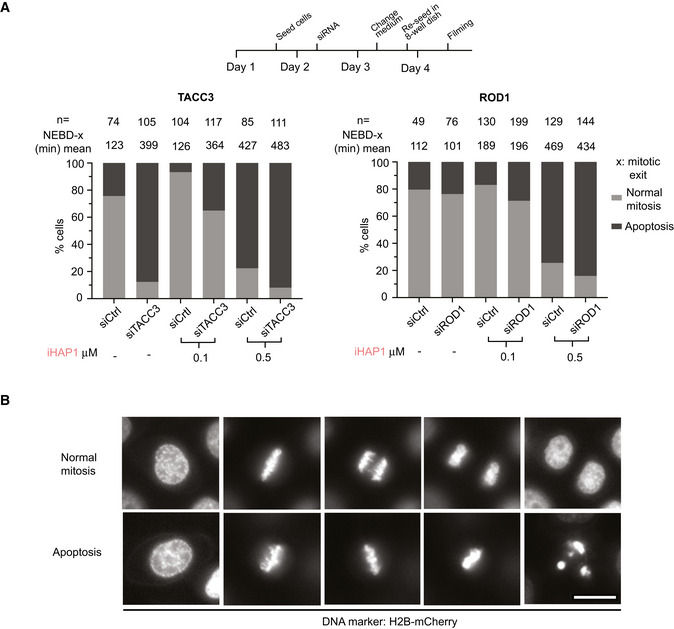
Depletion of TACC3 or Rod1 causes increased cell killing by iHAP1 during mitosis Measurement of time spent in mitosis by time‐lapse imaging taking into consideration whether cells exited mitosis or underwent cell death during mitosis. *n* indicates the number of cells analyzed from at least two independent experiments. Schematic of experimental protocol on the top. Cells were treated with iHAP1 when indicated at the concentration stated in the figure.Representative stills from time‐lapse imaging experiments depicting the two phenotypes. Scale bar represents 20 µm. Source data are available online for this figure.

We next analyzed mitotic fidelity in iHAP1‐treated cells and the impact of TACC3 and Rod1 removal. We used a HeLa cell line expressing fluorescent markers for tubulin and chromosomes and analyzed mitotic progression by time‐lapse microscopy (Fig 5A and B). We used low doses of iHAP1 to monitor possible synergistic effects and recorded whether cells successfully progressed through mitosis or died during a mitotic arrest. Even though depletion of TACC3 and Rod1 causes quite distinct effects on mitotic timing, the proportion of cells dying during an iHAP1‐induced mitotic arrest increased upon both TACC3 and Rod1 depletion (Fig 5A and B). This could explain the hypersensitivity to iHAP1 when TACC3 and Rod1 are depleted.

In summary, all our data show that iHAP1 is a microtubule poison.

### DT‐061 disrupts Golgi and ER

**Figure 6:**
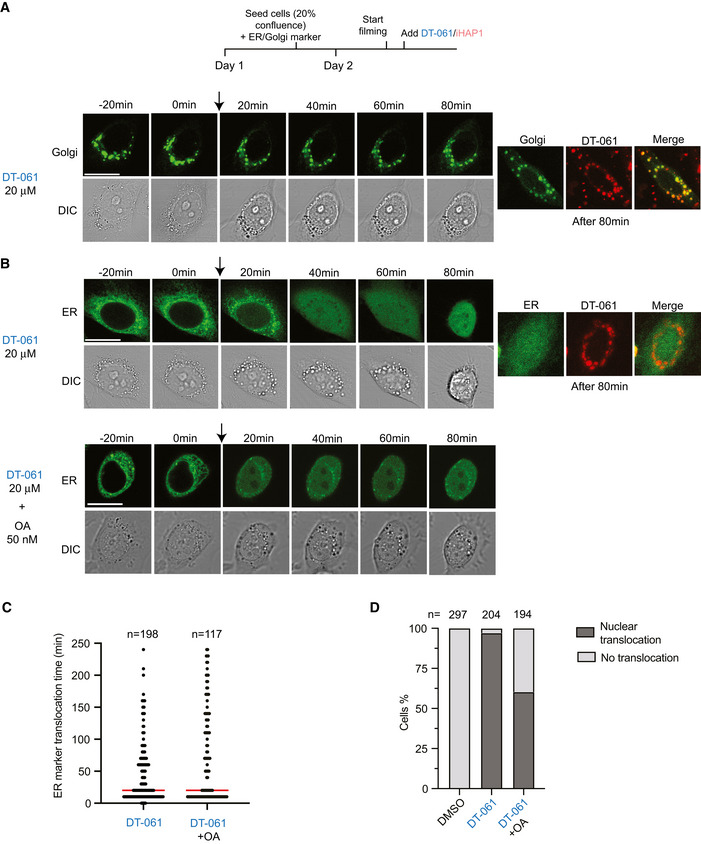
DT‐061 disrupts Golgi and ER structures in HeLa cells Time‐lapse imaging of HeLa cells expressing a marker staining the Golgi apparatus (green) (*n* = 87 of cells from 2 experiments analyzed all showing dispersal of marker). At time 0, DT‐061 was added. On the right‐hand side, the co‐localization of DT‐061 (red) and the Golgi marker is shown.As in (A) but with a marker for ER and either with the addition of DT‐061 alone or DT‐061 + okadaic acid (50 nM)Time required for ER signal translocation into the nucleus after drug treatment. Number of cells analyzed per condition shown on top and median indicated by red line.Percentage of cells with ER signal translocation into the nucleus after drug treatment. Number of cells analyzed per condition shown on top. Data information: (A–D) Experiments were repeated at least twice and stills show a representative cell for each treatment condition. Cells were treated with DMSO (Ctrl) or DT‐061 at the concentration stated in the figure. (A and B) Scale bar indicates 20 µm. Source data are available online for this figure.

**Figure EV5:**
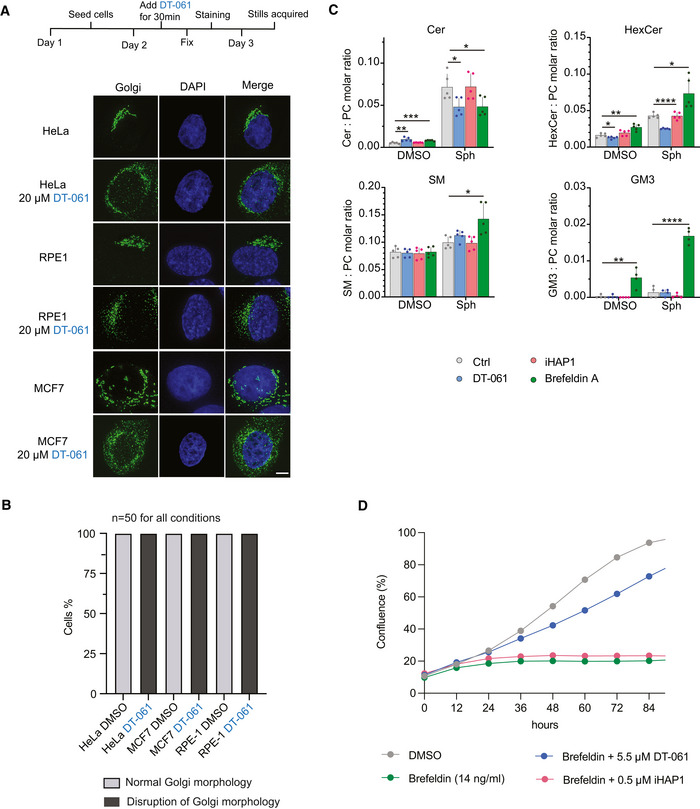
Analysis of DT‐061 effects on Golgi Immunofluorescence analysis of Golgi using an antibody against GM130. The indicated cell lines were treated with 20 µM DT‐061 for 30 min before fixation. Representative images shown. Scale bar 5 µm.Percentage of cells showing each phenotype after treatment with DT‐061. Number of cells analyzed per condition from a single experiment is shown.Molar quantities of sphingolipid classes detected in MCF7 cells after feeding with the precursor sphingosine or treatment with vehicle (DMSO). MCF7 cells were co‐treated with 15 μM DT‐061, 1.5 μM iHAP1, 3 µg/ml Brefeldin A or vehicle (DMSO). The determined molar quantities of sphingolipid classes were normalized to that of PC. The presented values represent averages of five independent experiments, and statistical analysis was performed using an independent *t*‐test. *P < 0.05, **P < 0.01, ***P < 0.001, ****P < 0.0001. Cer: ceramide, HexCer: hexosylceramide, PC: phosphatidylcholine and SM: sphingomyelin.Growth curves of HeLa cells treated with the indicated combinations and concentration of drugs. The results shown are representative of four independent experiments.

**Figure 7:**
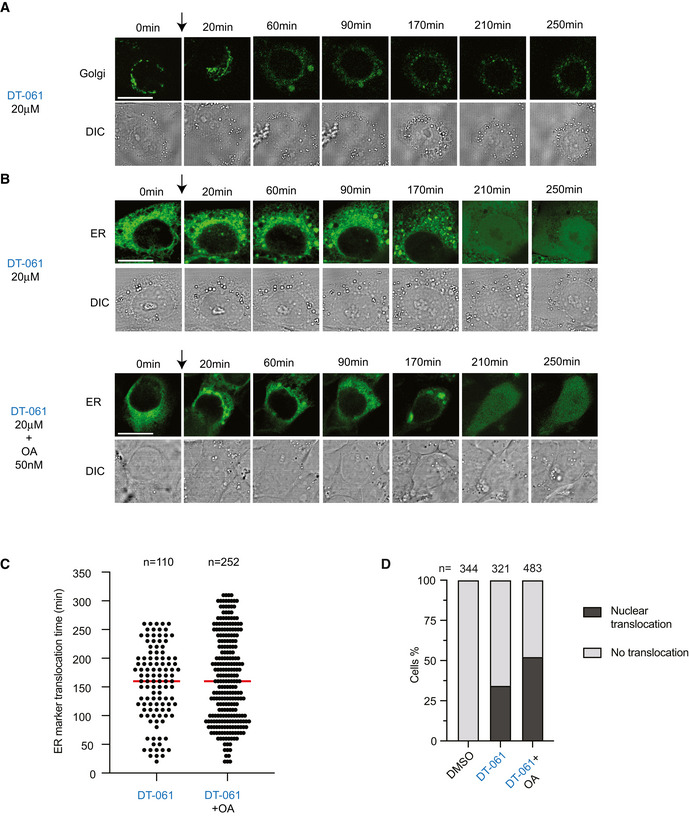
DT‐061 disrupts the structure of Golgi and ER in H358 cells A, BTime‐lapse imaging of H358 cells expressing a marker for the Golgi (A) or the ER (B). Cells were treated with DT‐061 at time 0. (B, low panel). For (A) 177 cells were analyzed from one experiment and all displayed dispersal of the Golgi marker. Arrow indicates addition of compound.CTime needed for ER translocation into the nucleus after drug treatment. Median (red line): 160 min. Number of cells analyzed per condition shown on top.DPercentage of cells with ER nuclear translocation after drug treatment. Number of cells analyzed per condition shown on top. Data information: (A–D) Experiments were repeated at least twice except filming of the Golgi marker which was done once. Stills show a representative cell for each treatment condition. Cells were treated with DMSO (Ctrl) or DT‐061 at the concentration stated in the figure. (A and B) Scale bar indicates 20 µm. Source data are available online for this figure.

The TRAPP complex is known to regulate the transport between ER and Golgi, while the NatC complex is required for N‐terminal acetylation of proteins (Polevoda & Sherman, 2003; Kim *et al*, 2016). Depletion of both TRAPP complex and NatC components has been shown to result in Golgi fragmentation (Behnia *et al*, 2004; Scrivens *et al*, 2009; Starheim *et al*, 2017). Given that CRISPR‐mediated removal of TRAPP and NAA complex components sensitized cells to DT‐061, we investigated Golgi integrity in cells treated with DT‐061. We used a live cell Golgi marker to investigate immediate effects upon DT‐061 addition. Moreover, since many tricyclic compounds are fluorescent at low nm wavelength, which is also the case for DT‐061 (Appendix Fig S9A), we could directly visualize the subcellular localization of the compound. Strikingly upon addition of 20 μM DT‐061 to HeLa cells, we observed a rapid disruption of the Golgi complex, which spread throughout the cytoplasm and disintegrated into large vesicle‐like structures that in a fraction of cells co‐localized with DT‐061 (Fig 6A, Movie EV5). We observed a similar effect on the Golgi complex in HeLa, RPE1 and MCF7 cells by immunostaining with an antibody against GM130, a marker for the cis‐Golgi (Fig EV5A–B). Using a live cell marker for the ER, we observed that this translocates to the nucleus approximately 20 min after addition of DT‐061 in HeLa cells (Fig 6B–C, Movie EV6). The ER marker is concentrated in the lumen of the ER through a localization signal and retention signal, and we use its nuclear translocation as a readout of ER function and integrity. The percentage of cells displaying translocation of the ER marker within the 5 h of recording was reduced to approximately 60% by 50 nM okadaic acid while the kinetics where unaffected (Fig 6C and D). The effects of DT‐061 on Golgi and ER markers in HeLa cells were not observed with 2 μM iHAP1 (Appendix Fig S9B). We next analyzed the effect of DT‐061 on the Golgi and ER markers in H358 cells. The addition of DT‐061 resulted in dispersal of the Golgi marker throughout the cytoplasm within approximately 60 min (Fig 7A and Movie EV7). In H358 cells expressing the ER marker, the addition of DT‐061 resulted in the formation of larger aggregates in the cytoplasm in the majority of cells followed by dispersal of these with subsequent nuclear translocation in a fraction of cells (Fig 7B–D, Appendix Fig S10 and Movie EV8). For the cells where there was no translocation of the ER marker, the aggregates often remained for the duration of the experiment or dispersed and reappeared. The addition of okadaic acid did not affect rates or extend of ER marker translocation, arguing that it is independent of effects on PP2A in this cell line. These results show that DT‐061 affects both Golgi and ER integrity, consistently with the chemogenic profile of the compound.

**Figure 8:**
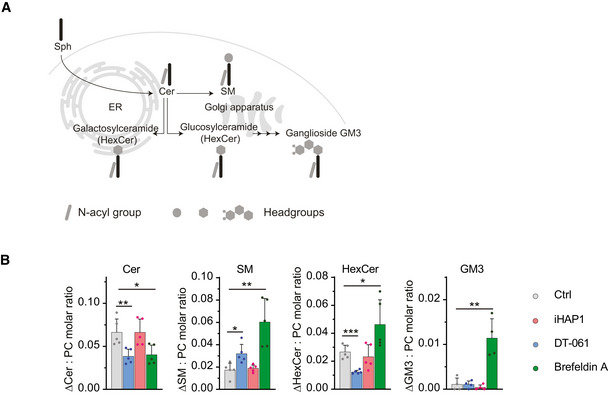
DT‐061 affects sphingolipid biosynthesis in Golgi and ER Schematic illustration of cellular sphingolipid biosynthesis in ER and Golgi.Increase in the molar quantity of sphingolipid classes assessed in MCF7 cells after feeding with the precursor sphingosine. MCF7 cells were co‐treated with 15 μM DT‐061, 1.5 μM iHAP1, 3 µg/ml Brefeldin A or vehicle (DMSO). The determined molar quantities of sphingolipid classes were normalized to that of PC. The presented values represent averages of five independent experiments, and statistical analysis was performed using an independent *t*‐test. *P < 0.05, **P < 0.01, ***P < 0.001. Cer: ceramide, HexCer: hexosylceramide, PC: phosphatidylcholine and SM: sphingomyelin. Source data are available online for this figure.

Considering the impact on Golgi and ER integrity, we analyzed if DT‐061 affects biosynthesis of sphingolipids as these biosynthesis pathways are tightly linked to these cellular structures (Fig 8A) (Holthuis & Menon, 2014). We fed breast carcinoma MCF7 cells with sphingosine, a precursor for sphingolipids, for 4 h and then conducted quantitative mass spectrometry‐based shotgun lipidomics to measure the induced changes in the levels of ceramide, sphingomyelin (SM) and glycosphingolipids hexosylceramide (isomers glucosyl‐ and galactrosylceramide) and ganglioside GM3. This was done in the presence of 15 μM DT‐061, 1.5 μM iHAP1 and, as a control, Brefeldin A, which is known to inhibit vesicular transport between ER and Golgi, eventually causing the collapse of Golgi into ER (Dascher & Balch, 1994). In all our experiments, iHAP1‐treated cells behaved similarly to the control condition, indicating that iHAP1 does not affect the biosynthesis pathways of sphingolipids analyzed here. For DT‐061 and Brefeldin A, we observed a reduction in conversion of sphingosine to ceramide, a reaction occurring on the cytosolic side of the ER (Figs 8B and EV5C and Appendix Figs S11 and S12). Once transferred to the Golgi, ceramide can be modified into SM or glycosphingolipids through addition of various headgroups. Conversion of ceramides to SM was increased by both DT‐061 and Brefeldin A, while conversion to glycosphingolipids was specifically inhibited by DT‐061. In contrast, we observed enhanced ceramide to glycosphingolipid conversion in the presence of Brefeldin A. Interestingly, DT‐061 and Brefeldin A had antagonistic effects on the viability of cells (Fig EV5D).

Collectively, our work shows that DT‐061 has a clear and immediate effect on Golgi and ER integrity which impacts lipid biosynthesis pathways occurring at these cellular structures.

## Discussion

PP2A dampens the activity of oncogenic signaling pathways, and for this reason there is a considerable interest in strategies able to activate PP2A holoenzymes. Here, we extensively analyzed two tricyclic heterocyclic compounds that have been claimed to activate specific PP2A‐B56 complexes to inhibit cellular proliferation (Leonard *et al*, 2020; Morita *et al*, 2020). Collectively, our work argues that none of these compounds have a specific effect on PP2A‐B56 and that their antiproliferative effects are likely unrelated to PP2A‐B56 activation.

The molecular targets of iHAP1 are microtubules, and this compound has a clear effect on microtubule stability both *in vitro* and *in vivo*. This has already been documented by a previous study characterizing iHAP1 and related compounds (Prinz *et al*, 2011). Why this effect was not observed by Morita *et al* (2020) in their *in vitro* experiments is unclear to us. The fact that iHAP1 is a microtubule poison is consistent with the cellular phenotypes observed in the present work and by Morita *et al* (2020), namely a strong mitotic arrest. However, we disagree on the molecular mechanism behind the mitotic arrest. Morita *et al* (2020) argued that the effect is mediated through PP2A‐B56ε dephosphorylation of MYBL2, which results in a transcriptional deregulation of mitotic proteins. We see an immediate effect of iHAP1 on microtubule dynamics in interphase and mitotic cells, thus arguing against any transcriptional effects.

The effect of DT‐061 on Golgi and ER integrity is striking and fully consistent with the chemogenetic profile of the compound established by our CRISPR screen. Depletion of TRAPP and NatC components is known to affect Golgi integrity, which in turn sensitizes these cells to further perturbation of the Golgi and ER by DT‐061. At present, we do not know the direct molecular target of DT‐061, but we favor the idea that it is a lipid‐constituting part of the Golgi or ER. This would be consistent with the hydrophobic nature of DT‐061 and the fact that we see DT‐061 forming small structures in cells attracting the Golgi marker. Tricyclic heterocyclic compounds can have a profound impact on lipids, and indeed methylene blue, which belongs to this class of molecules, has been used to stain the Golgi in cells (Gatenby & Moussa, 1948; Wesolowska *et al*, 2011; Mahajan & Mahajan, 2013; Daniel *et al*, 2015; Tummino *et al*, 2021). We acknowledge the extensive studies conducted by Leonard *et al* (2020) to analyze the interaction between DT‐061 and PP2A‐B56α complexes. The biochemical experiments in Leonard *et al* (2020) focused on the stabilization of PP2A complexes by DT‐061 which might not reflect direct binding. We were unable to recapitulate a stabilizing effect on the PP2A‐B56α holoenzyme as observed by Leonard *et al* (2020) in a large panel of *in vitro* or *in vivo* assays. We cannot rule out that very specific experimental conditions are required to see this. The cryo‐EM structure of PP2A‐B56α‐DT‐061 argues for a direct binding of DT‐061, but our analysis of this structure argues that the assignment of density is not unambiguous. Our comparison of reported crystal structures of PP2A‐B56γ complexes with the cryo‐EM structure of DT‐061 bound to PP2A‐B56α indicates that the flexible C‐terminal tail of the catalytic subunit could account for the density ascribed to DT‐061. Despite this disagreement, our extensive analysis reveals that DT‐061 affects Golgi and ER function and integrity and the chemogenetic profile of DT‐061 supports that this is the major mechanism of cellular toxicity.

Collectively, our work argues that the PP2A‐activating properties of iHAP1 and DT‐061 and possibly other tricyclic compounds should be carefully reconsidered. Previous literature using such compounds to make claims on PP2A biology should also be revisited.

## Materials and Methods

### Compounds

We obtained iHAP1 from Enamine (Cat#: Z56843374) and DT‐061 (Cat#: HY‐112929) from either MedChemExpress or as a gift from Goutham Narla. Perphenazine was purchased from Sigma‐Aldrich/Merk (Cat#P6402). Okadaic acid was bought from Enzo Life Sciences (Cat#ALX‐350‐003). Compounds were dissolved according to instructions and stored as single‐use aliquots. Their identity was confirmed by mass spectrometry.

### Antibodies

Following primary antibodies were used at the indicated dilutions: Myc tag (#ab32, 1:1,000, Abcam), PPP2R1A (#2041, 1:2,500, Cell Signaling Technology), B55α (#5689, 1:2,500, Cell Signaling Technology), B56α (#610615, 1:3000, BD Biosciences), B56δ (#5687, 1:2,500, Cell Signaling Technology), B56ε (#PA5‐17981, 1:2,000, Thermo Fisher Scientific), PP2AC Methyl (Leu 309) (#828801, 1:3,000, BioLegend), PP2AC (#05‐421, 1:4,000, Millipore/Merk), actin, (#sc‐47778 HRP, 1:5,000, Santa Cruz Biotechnology), TRAPPC2L (#HPA041714, 1:2,000, Sigma‐Aldrich), TACC3 (sc‐376883, 1:1,000, Santa Cruz Biotechnology), ROD1 (1:300, a kind gift from Reto Gassmann’s lab), GM‐130 (#610822, 1:500, BD Biosciences), Tubulin (#T9026, DM1A, 1:2,000, Sigma‐Aldrich).

### Cell culture methods

Human cell cultures were maintained at 37°C in a humidified 5% CO_2_ environment. HeLa cells (ATCC CCL‐2), H358 cells (ATCC CRL‐5807), U2OS (ATCC HTB‐96) c and HEK‐293T (ATCC CRL‐3216) cells were cultured in the appropriate medium supplemented with the necessary nutrients as indicated by the American Type Culture Collection, Manassas, Virginia, USA. Cells were not authenticated or tested for mycoplasm during this work. Cell media were supplemented with 10% Fetal Bovine Serum (FBS) (Thermo Fisher Scientific) and 1% Penicillin‐Streptomycin 10,000 U/ml (Thermo Fisher Scientific). Media used for RNAi experiments was devoid of Penicillin‐Streptomycin.

CRISPR/Cas9‐inducible knockout cell lines were cultured in presence of tetracycline‐free FBS (Thermo Fisher Scientific). To activate Cas9 activity, cells were induced with Doxycycline at a concentration of 1 µg/ml for 4 days. Downregulation/knockout of the protein of interest was assessed by Western blotting.

Parental U2OS and U2OS cell lines stably expressing H2B‐GFP and mCherry‐α tubulin (gift from S. Geley, Innsbruck Medical University, Austria) (Barisic *et al*, 2010), EB1‐GFP (Vinopal *et al*, 2012) (gift from P. Draber, IMG ASCR, Prague, Czech Republic) were grown in Dulbecco’s Modified Eagle Medium (DMEM; Gibco) supplemented with 10% fetal bovine serum (FBS; Invitrogen) at 37°C with 5% CO_2_.

U2OS EB1‐GFP cells were treated with DT‐061 and iHAP1 20 min before imaging. To arrest cells in metaphase, cells were incubated with the Anaphase‐Promoting Complex/Cyclosome (APC/C) inhibitors Apcin (20 µM) and a cell permeable tosyl‐L‐arginine methyl ester (proTAME) (10 µM) for 2 h before the addition of DT‐061 or iHAP1. DNA was labeled by adding 20 nM SiR‐DNA (Spirochrome) 2 h prior to live‐cell imaging.

### *In vitro* phosphatase assay

PP2A holoenzymes were purified from inducible HeLa cell lines stably expressing FLAG‐Venus tagged B55α, B56α and B56ε regulatory subunits, respectively, which were previously generated in the lab (Kruse *et al*, 2013; Hertz *et al*, 2016). Cells were grown in presence of 10 ng/ml Doxycycline for 48 h and washed three times in 50 ml ice‐cold PBS. A total of ~ 8 × 10^7^ cells were lyzed in 2 ml lysis buffer (20 mM Tris HCl pH 7.4, 150 mM NaCl, 1% Triton‐X 100 supplemented with protease inhibitor Complete, Roche). Lysates were rotated at 4°C for 20 min and subsequently centrifuged at 20,000 *g* for 30 min. Anti‐FLAG M2 Affinity gel (Sigma‐Merck) was equilibrated in lysis buffer and gently shaken at 4°C for 3 h with the lysate. Beads were subsequently washed two times in lysis buffer and four times in TBS/0.1% Triton‐X 100, followed by one wash in TBS. Prior to elution, beads were washed one time in elution buffer (20 mM Tris–HCl pH 7.4, 150 mM NaCl, 1 mM MnCl_2_). Elution of the co‐immunoprecipitated complex was performed by gentle shake of the beads at 4°C in elution buffer supplemented with 3X FLAG peptide (Sigma‐Aldrich) for 30 min. Glycerol and DTT were added at a 25% and 1 mM final concentration, respectively, before storing the samples at −80°C. Non‐stick tubes were used during the purification and for storage. Purity of enzymatic preparations was assessed by colloidal staining (NOVEX Colloidal Blue Stain Kit, Invitrogen) and mass spectrometry. No other protein phosphatases were detected in the preparations. Titration curves were performed using different amounts of enzyme (2–10 µl).

*In vitro* reactions were performed in non‐stick tubes. Two microliters of enzyme were incubated with 1 mM basic (WRRA(pT)VA), acidic (WSGDD(pT)IVD), BRCA2 S1106 WT (P(pS)QKAEITELSTILEESGSQW) or BRCA2 S1106 2A (P(pS)QKAEITEASTALEESGSQW) peptide substrate together with 20 µM DT‐061 or 10 µM iHAP1 in phosphatase buffer (50 µM Tris–HCl pH 7.4, 0.3 mM NaCl and 2 mM MnCl_2_) for 15 min at 30°C. Peptides were purchased from Peptide 2.0 Inc (Chantilly, VA, USA). Inorganic phosphate release was assessed using the PiColorLock Phosphate Detection System (Expedon) according to manufacturer's instructions.

### Protein production and purification

GST‐PPP2R1A was expressed in *E. coli* and cells lyzed in buffer L (50 mM Tris–HCl pH = 7.5; 300 mM NaCl, 10% glycerol, 0.5 mM TCEP). Following lysis and clarification of the extract, it was loaded on a glutathione column and washed with buffer L. The fusion protein was eluted with buffer L containing 20 mM reduced glutathione and GST tag removed by TEV cleavage. The GST was removed by loading the TEV cleavage reaction on a glutathione column. Untagged PPP2R1A was further purified on a Superdex 200 16/60 equilibrated with buffer G (50 mM NaP pH = 7.5, 150 mM NaCl, 5% glycerol, 0.5 mM TCEP) and peak fractions were pooled. The protein was analyzed by LC‐MS.

PP2A‐B56α for *in vitro* phosphatase assays was reconstituted in HEK293 cells by co‐expressing Strep‐B56α, His‐PP2ACA and MBP‐PPP2R1A. The cell pellet was lyzed in 100 mM Tris pH = 8.0, 150 mM NaCl, 10% glycerol, 0.5 mM TCEP and the complex purified by a Strep‐tag affinity column, and the peak fractions were run on GF200 10/30 column and collected and frozen.

### NMR

1D‐^1^H‐NMR spectra with water suppression (bruker‐pulseprogram: zgesgp) were recorded at 25°C on a Bruker Avance III HD spectrometer equipped with a 5 mm TCI Cryoprobe H‐C/N‐D. The final protein concentration was 20 µM in all samples, and the compounds were added to a final concentration of 200 µM from a d_6_‐DMSO stock. The buffer composition in all samples were 116 mM KCl, 2.3 mM Na_2_HPO_4_, 1.7 mM KH_2_PO_4_, 10% d_6_‐DMSO and 5% D_2_O.

### Isothermal titration calorimetry (ITC)

Prior to ITC experiments, the recombinant PPP2R1A protein was extensively dialyzed against ITC buffer, 100 mM sodium phosphate pH 7.5, 150 mM NaCl, 0.5 mM TCEP. After dialysis, the ITC buffer was supplemented with either 5% or 10% DMSO (Sigma‐Aldrich, (#34943)) before running the ITC experiments. Both DT‐061 and iHAP were dissolved in the same dialysis buffer from an initial stock of 1 mM in pure DMSO to a final concentration of 20 μM and adjusting the percentage of DMSO to 5% and 10% with pure DMSO. Perphenazine was dissolved in the same dialysis buffer from an initial stock of 8 mM in pure DMSO to a final concentration of 400 μM. All ITC experiments were performed on an Auto‐iTC200 microcalorimeter (MicroCal‐Malvern Panalytical, Malvern, UK) at 25°C. The protein was loaded into the syringe at 300 μM and titrated into the calorimetric cell containing the DT‐061 or iHAP1 solutions at 20 μM. Control experiments with the protein injected in the sample cell filled with buffer were carried out under the same experimental conditions (Fig EV1). Control experiments with perphenazine injected in the sample cell filled with buffer were carried out under the same experimental conditions. In all assays, the titration sequence consisted of a single 0.4 μl injection followed by 19 injections, 2 μl each, with 150 s spacing between injections to ensure that the thermal power returns to the baseline before the next injection. The stirring speed was 750 rpm and the reference power set at 8 μcal/s. The heats evolved after each ligand injection were obtained from the integral of the calorimetric signal.

### Differential scanning fluorimetry (nanoDSF)

The thermal stability of PPP2R1A in both NMR and ITC buffers with different percentage of DMSO (from 0% to 40%) was evaluated by following the changes in the intrinsic fluorescence ratio (350/330 nm) as a function of the temperature. The experiments were run in a Prometheus NT.48 nanoDSF (NanoTemper Technologies). Ten microliters of the protein samples at 10 μM were measured in triplicates with a temperature ramp set to 1°C/min from 20°C to 80°C using standard treated capillaries (NanoTemper Technologies (#PR‐C002)). The PR. ThermControl v2.1.6 software (NanoTemper Technologies) was used for data acquisition and data analysis.

### Mass photometry (MP)

Mass photometry experiments was performed on a Refeyn One^MP^ (Refeyn Ltd) mass photometer at room temperature. Measurements were run in triplicates diluting the protein sample at 50 nM concentration in NMR buffer loaded on a gasket (Grace BioLabs reusable CultureWell™ gaskets, Merck (#GBL103250)) mounted on a glass microscope coverslip (Marienfeld (#0107222)). The molecular weight was obtained by contrast comparison with known molecular weight mass standard calibrants (NativeMark™ Unstained Protein Standard, Thermo Fisher Scientific, (#LC0725)) measured on the same buffer and on the same day. Movies were recorded using AcquireMP (Refeyn Ltd, version 2.3.2), with a regular field of view and exposure time of 0.95 ms for the acquisition camera settings. A total of 6,000 frames were recorded over a duration of 1 min. Movies were processed and analyzed with the DiscoverMP software (Refeyn Ltd, version 2.3.0) provided by the instrument manufacturer.

### PP2A‐B56α reconstitution for MP measurements

The strategy for production and purification of the PP2A(B56α) holocomplex was based on the work published by Cho and Xu (2007).

#### Expression and purification of Aα and B56α subunits

Both PPP2R1A and PPP2R5A were cloned into pGEX‐6P‐1 vectors. PPP2R5A was amplified by PCR from a MegaMan Human Transcriptome Library (Stratagene).

Both Aα and B56α subunits were expressed separately in *E. coli* BL21 Rosetta cells. Cells were grown in Super Broth media at 37°C, and the expression was induced overnight at 18°C with 0.35 mM IPTG when OD_600_ was ≈ 1.0. Cultures were centrifuged at 4,000 *g* for 10 min at 4°C and resuspended in lysis buffer (20 mM Tris–HCl pH 8.0, 500 mM NaCl, 2 mM dithiothreitol [DTT]) supplemented with 10 μg/ml DNase and 1 mM PMSF. Cells were disrupted by sonication and the supernatant was recovered by centrifuging the lysate at 22,000 rpm for 50 min at 4°C. The supernatant was incubated with 10 ml GSH beads (GE Healthcare^®^) for 2 h at 4°C. GSH beads were subsequently washed with 300 ml of lysis buffer, 200 ml of high salt buffer (20 mM Tris–HCl pH 8.0, 1,000 mM NaCl, 50 mM KCl, 10 mM MgCl_2_, 2 mM ATP, 2 mM DTT) and 200 ml of lysis buffer. The proteins were cleaved by addition of 2 mg of 3C protease while proteins were still bound to the beads. Eluted protein was concentrated using an Amicon Ultra 15 10 kDa MWCO centrifugal unit (Millipore^®^) prior to size exclusion chromatography.

The protein was applied to a HiLoad Superdex 200 pg column pre‐equilibrated with 20 mM Tris–HCl pH 8.0, 500 mM NaCl, 5% (V/V) glycerol, 2 mM DTT. Fractions containing pure protein were analyzed by SDS–PAGE, pooled and snap frozen.

#### Expression and purification of Cα subunit

Bacmids containing His‐tagged Cα (D88N) subunit were generated using the MultiBac technology. Sf9 cells were used to obtain highly infective baculoviruses (V_2_). For large‐scale protein production, Hi5 cells grown at a density of 2 × 10^6^ cells/ml were infected with V_2_ (1:25 ratio) for 48 h. Cultures were centrifuged at 800 *g* for 15 min and pellets were resuspended with 20 mM Tris–HCl pH 8.0, 250 mM NaCl, 35 mM imidazole, 2 mM β‐mercaptoethanol. Sonication was used to disrupt the cells, and cell debris was pelleted by centrifugation at 22,000 *g* for 90 min at 4°C. The supernatant was incubated with 3‐ml Ni^2+^‐NTA resin (GE Healthcare^®^) and incubated for 3 h at 4°C. Beads were recovered and washed with 200 ml of lysis buffer, 100 ml of high salt buffer (20 mM Tris–HCl pH 8.0, 1,000 mM NaCl, 35 mM imidazole, 50 mM KCl, 10 mM MgCl_2_, 2 mM ATP, 2 mM β‐mercaptoethanol) and 200 ml of lysis buffer. The Cα subunit was eluted in 20 mM Tris–HCl pH 8.0, 500 mM NaCl, 350 mM imidazole, 2 mM β‐mercaptoethanol.

#### PP2A(B56α) holocomplex reconstitution

Pure PP2A Aα and B56α subunits and partially purified Cα subunit were mixed at a 1:1:1 molar ratio and dialyzed overnight at 4°C in buffer containing 20 mM Tris–HCl pH 8.0, 150 mM NaCl, 5% (V/V) glycerol and 2 mM DTT. The dialyzed sample was applied onto a 5‐ml HiTrap Q HP anion exchange chromatography column (GE Healthcare^®^) previously equilibrated with the dialysis buffer. The PP2A(B56α) holocomplex was eluted using a gradient of buffer containing 20 mM Tris–HCl pH 8.0, 1,000 mM NaCl, 5% (V/V) glycerol and 2 mM DTT. Eluted protein was concentrated using an Amicon Ultra 15 10 kDa MWCO centrifugal unit (Millipore^®^) prior to size exclusion chromatography.

Sample was applied to a HiLoad Superdex 200 pg column pre‐equilibrated with 20 mM Tris–HCl pH 8.0, 250 mM NaCl, 5% (V/V) glycerol, 2 mM DTT. Fractions containing the PP2A(B56α) holocomplex protein were analyzed by SDS–PAGE, pooled and snap frozen.

### Immunoprecipitation

HEK‐293T cells stably expressing inducible myc‐PP2A/C (catalytic) (Nasa *et al*, 2020) and HeLa cells stably expressing inducible FLAG‐myc‐PPP2R1A (scaffold) (generated for this study), FLAG‐Venus‐PP2A‐B55α, ‐B56α (described in (Hertz *et al*, 2016)) and ‐B56ε (generated for this study) subunits were induced with 10 ng/ml Doxycycline 48 h before immunoprecipitation. After treatment with DT‐061 20 µM, iHAP1 2 µM or DMSO for 30 min and 2 h, respectively, cells were lyzed in low salt lysis buffer (50 mM NaCl, 50 mM Tris pH 7.4, 1 mM EDTA, 0.1% NP‐40, 1 mM DTT) supplemented with complete protease inhibitor (Roche). Lysates were rotated at 4°C for 20 min prior to maximum speed centrifugation at 4°C for 30 min. Clear protein lysate was transferred to a new, non‐stick prechilled reaction tube to perform immunoprecipitation. Myc‐Trap Agarose affinity reagent (Chromotek) was used to immunoprecipitated myc‐PP2A/C subunit and FLAG‐myc‐PPP2R1A, and Anti‐FLAG M2 Affinity gel (Sigma‐Merck) for PP2A regulatory subunits B55α, B56α and B56ε. Beads were equilibrated in lysis buffer and then rotated for 1 h in the protein lysate of interest at 4°C. Beads were subsequently washed three times in lysis buffer, and bound complexes were eluted using 4× Lämmli sample buffer (Biorad). Samples were boiled at 95°C and resolved through SDS–PAGE prior to Western blotting or analysis by quantitative mass spectrometry as described in the label‐free LC‐MS/MS analysis section. Experiments were performed in triplicate.

### Label‐free LC‐MS/MS analysis

*PIB pulldowns* were analyzed on a Q‐Exactive Plus quadrupole Orbitrap mass spectrometer (ThermoScientific) equipped with an Easy‐nLC 1000 (ThermoScientific) and nanospray source (ThermoScientific) as previously described (Lyons *et al*, 2018). Raw data were searched using COMET (release version 2014.01) in high‐resolution mode (Eng *et al*, 2013) against a target‐decoy (reversed) (Elias & Gygi, 2007) version of the human proteome sequence database (UniProt; downloaded 2/2020) with a precursor mass tolerance of ± 1 Da and a fragment ion mass tolerance of 0.02 Da, and requiring fully tryptic peptides (K, R; not preceding P) with up to three mis‐cleavages. Static modifications included carbamidomethylcysteine and variable modifications included oxidized methionine. Searches were filtered to a < 1% FDR at the peptide level. Quantification of LC‐MS/MS spectra was performed using MassChroQ (Valot *et al*, 2011) and the iBAQ method (Schwanhausser *et al*, 2011). PPP subunit abundances were normalized across all samples by quantile normalization. Statistical analysis was carried out in R statistical software (version 4.0.5) in conjunction with R studio (version 1.3) (Team, 2019) by two‐tailed Student's *t*‐test.

*B56α, B56ε and B55α* purifications were analyzed on an Orbitrap Fusion Lumos mass spectrometer (ThermoScientific) equipped with an Easy‐nLC 1200 (ThermoScientific), searched, filtered and quantified as described above.

*PP2AC pulldowns* were analyzed on an Orbitrap Fusion Lumos mass spectrometer (ThermoScientific) equipped with an Easy‐nLC 1200 (ThermoScientific), searched, filtered and quantified as described above. PP2Ac abundance was normalized across all samples to be equal. Statistical analysis was carried out in R statistical software (version 4.0.5) in conjunction with R studio (version 1.3) (Team, 2019) by two‐tailed Student's *t*‐test.

### Size exclusion of cell extracts

Cells were harvested and washed once with PBS and then resuspended in 5 volumes of lysis buffer (130 mM NaCl, 25 mM Tris pH = 7.4, 5 mM b‐mercaptoethanol, 0.1% NP‐40, protease inhibitors) and sonicated using 10 sonication cycles. For the DT‐061‐treated samples, the lysis buffer contained 20 μM DT‐061. The cell lysate was clarified by centrifugation at 20,000 *g* for 30 min at 4 degrees and 500 μl immediately applied to a Superdex200 10/300 gl (GE Healthcare) column equilibrated with buffer G (130 mM NaCl, 25 mM Tris pH = 7.4, 5 mM b‐mercaptoethanol). For DT‐061‐treated samples, buffer G contained 2 μM DT‐061. The flowrate was 0.5 ml/min, and 0.5 ml fractions were collected and analyzed by Western blotting using a Licor Clx instrument and Image studio software. Quantification of band intensities was performed with Image Studio.

### Structural analysis

The corresponding cryo‐EM map, structure factors and PDBs for 6NTS (Leonard *et al*, 2020), 2IAE (Cho & Xu, 2007), 2NNP (Xu *et al*, 2006) and 3FGA (Xu *et al*, 2009) were downloaded from the PDB and EMDB databases. Structure factors and PDBs were employed to calculate the maps of 2IAE and 3FGA. The maps and the PDBs were later superimposed on the catalytic subunit of 6NTS using phenix_superpose_maps in the Phenix suite (Adams *et al*, 2010). The superposition shows that the tail of the catalytic subunit can be also traced in the cryo‐EM map, similarly to 2IAE, 2NNP and 3FGA. Maps were visualized with Chimera (Pettersen *et al*, 2004) and Coot (Emsley *et al*, 2010) and refined in phenix (Adams *et al*, 2010).

### CRISPR‐Cas9 knockout screens

Viral particles of the LCV2:TKOv3 sgRNA library were produced as previously described (Hart *et al*, 2017). RPE1‐hTERT Flag‐Cas9 TP53^−/−^ cells were transduced with a MOI of 0.3 at a coverage of > 350‐fold sgRNA representation, which was maintained throughout the screen at each cell passage point. Twenty‐four hours after transduction, transduced cells were selected for 24 h with 25 µg/ml puromycin. Cells were treated with trypsine and reseeded in the same plates while maintaining puromycin selection for another 24 h. Three days after transduction, which was considered the initial time point (T0), cells were pooled and passaged while cell pellets of two replicates of 3.5 × 10^7^ cells were frozen for downstream processing. Cells were passaged after 3 and 9 days after transduction, respectively, which was considered T6; cells were split into technical duplicates of either mock‐treated conditions or treated with DT‐061 (5.5 µM) or iHAP1 (0.5 µM) equivalent to pre‐determined LD20 concentrations in uninfected RPE1‐hTERT Flag‐Cas9 TP53^−/−^ cells treated for 12 days. Cells were subcultured every 3 days (T9, T12 and T15) in medium with or without drugs until the final time point at T18 at which cell pellets from 3.5x10^7 cells were frozen from each replicate. Genomic DNA from cells collected at T0 and T18 was isolated as previously described (Chen *et al*, 2015) and sgRNA sequences amplified by PCR using Q5 Mastermix Next Ultra II (New England Biolabs, Cat# M5044L) with the following primers: LCV2_forward: 5'‐GAGGGCCTATTTCCCATGATTC‐3' and LCV2_reverse: 5'‐GTTGCGAAAAAGAACGTTCACGG‐3'. This was followed by a second PCR reaction containing i5 and i7 multiplexing barcodes, and final gel‐purified products were sequenced on Illumina NextSeq500. Fastq files were generated using bcl2fastq v2.19.1, and reads were trimmed to 20 bp using cutadapt 1.18 (Martin, 2011) removing a variable number of bp at start and end depending on the size of the primer stagger. MAGeCK 0.5.8 (Li *et al*, 2014) was used to assign the trimmed reads to the guides in the TKOv3 library and create the count matrix. Gene scores (normZ values) were estimated from the count matrix using the drugZ algorithm (Colic *et al*, 2019). To assess data quality, we generated precision‐recall curves, calculated by the BAGEL.py “pr” function (Hart & Moffat, 2016) using the core essential (CEGv2.txt) and nonessential (NEGv1.txt) gene lists from https://github.com/hart‐lab/bagel, comparing T0 to T18 for mock‐treated cells.

### Tubulin polymerization assay

Assembly competent tubulin was purified from porcine brain as described before (Castoldi & Popov, 2003). Turbidity‐based microtubule polymerization assay was performed as described before (Mirigian *et al*, 2013). Briefly, indicated concentrations of iHAP1 and DT‐061 or DMSO (vehicle control) were mixed with free tubulin (2 mg/ml final concentration), and microtubule assembly was induced by the addition of 1 mM GTP and 10% glycerol in BRB80 buffer (80 mM PIPES, pH 6.8, 1 mM MgCl_2_ and 1 mM EGTA) at 37°C. Paclitaxel (taxol), a polymerization enhancer, and nocodazole, a microtubule depolymerizer, were used as internal controls for the polymerization reaction. Microtubule polymerization was monitored by measuring the change in absorbance (340nm) using SpectraMax^®^ iD3 (Molecular Devices) microplate reader in 30 s intervals over 60 min.

### Live‐cell imaging

Time‐lapse imaging was performed as described before (Liao *et al*, 2019). Briefly, cells were cultured in 35 mm glass‐bottomed dishes (14 mm, No. 1.5, MatTek Corporation), and imaging was performed in an environment controlled chamber (37°C with controlled humidity and 5% CO_2_ supply), using a Plan‐Apochromat DIC 63x/1.4NA oil objective mounted on an inverted Zeiss Axio Observer Z1 microscope (Marianas Imaging Workstation from Intelligent Imaging and Innovations Inc. (3i), Denver, CO, USA), equipped with a CSU‐X1 spinning‐disk confocal head (Yokogawa Corporation of America) and four laser lines (405 nm, 488 nm, 561 nm and 640 nm). Image detection was performed using an iXon Ultra 888 EM‐CCD camera (Andor Technology).

Mitosis in U2OS‐H2B‐GFP/mCherry‐α tubulin cells was imaged using fifteen 1 μm‐separated z‐planes collected every 2 min. Microtubule dynamics were monitored by imaging U2OS EB1‐GFP cells every 500 ms. Two to five EB1 comets per cell were tracked manually using ImageJ. In both experiments, iHAP1 or DT‐061 were added 20 min before imaging.

### Immunofluorescence staining

Cells were seeded on coverslips 24 h before immunofluorescence staining. Cells were then fixed in 4% paraformaldehyde (PFA) (Thermo Fisher Scientific) solution for 10 min and subsequently permeabilized with 0.2% triton X‐100 (Sigma‐Aldrich) for 5 min, washed once in PBS and blocked for 1 h in 3% albumin (Sigma‐Aldrich) in PBS. Subsequently, cells were incubated with an antibody against GM‐130 (see antibody section) for 1 h. Three washes with PBS followed, each for 5 min. Species‐specific secondary antibody was then incubated for 30 min in the dark. DNA was counterstained with DAPI solution (#62248, 1:2,000, Thermo Fisher Scientific) diluted in PBS, together with the secondary antibody (see antibody section). The coverslips were subsequently washed three times with PBS and briefly rinsed with 100% reagent grade ethanol. Coverslips were then mounted in Vectashield antifade mounting medium (Vector Laboratories) and were analyzed by fluorescence microscopy. Stills with z‐stacks 200 nm apart were taken using the DeltaVision Elite system microscope (GE Healthcare), 100× oil immersion objective, 1 × 1 bin. The same settings were applied for all immunostaining stills: DAPI 5% laser power (0.025 exposure), FITC 10% laser power (0.2 exposure), followed by deconvolution using Softworx.

U2OS cells were treated with the APC/C inhibitors Apcin (20 µM) and proTAME (10 µM) for 2 h followed by DT‐061 or iHAP1 for 20 min, fixed and stained as described before (Steblyanko *et al*, 2020). Mouse anti α‐tubulin was used as primary antibody (see antibody section).

### Transient siRNA transfection

Silencer Select Pre‐designed siRNA oligomers were purchased from Life Technologies LTD. Following antisense oligos were transfected as a pool as described below: TRAPPC2L (#s28534): 1: GCAUGUUCCGGAAGCUACA; 2: AGCCCUUCGAGACAACGAA; 3: AGGUGAAGUUUGUCAUGGU. ROD1 (#s18778): 1: GGAGAAUGUCUGUAGCGAA; 2: CCGUGGAUCUAGAAUAUCA; 3: CCACCAUAGUGUUCGAAU. TACC3 (#s20471): 1: CACCUCGACUGGGACAAAA; 2: GCAUGCACGGUGCAAAU. A non‐targeting oligo against Luciferase was used as control: CGUACGCGGAAUACUUCGA. The oligos were briefly centrifuged and resuspended in nuclease‐free water according to the manufacturer’s instructions. Seeding of cells was performed at the same time of transfection. Two hundred microliters Opti‐MEM medium (Gibco) was mixed with 2 μl of a 1 nMol stock of the oligonucleotides and briefly resuspended. Four microliters of Lipofectamine RNAiMAX Transfection Reagent (Thermo Fisher Scientific) were added to the solution and incubated at room temperature for 15 min. Subsequently, the mixture was added dropwise on the fresh‐seeded cells in a ~ 10 cm^2^ culture dish area in 1.8 ml Opti‐MEM medium and incubated for 72 h.

### Transient siRNA transfection and live imaging

HeLa cells stably expressing Tubulin‐mVenus, H2B‐mCherry (gift from Barr Lab) were transiently transfected with the following antisense oligos: ROD1 (5′ GGAAUGAUAUUGAGCUGCUAACAAA 3′; Thermo Fisher Scientific) and TACC3 (5’ GAGCGGACCUGUAAAACUA 3’), and a non‐targeting oligo against Luciferase was used as control (Sigma, Custom order). RNAi Max (Invitrogen) was used according to manufacturer's instructions.

Live cell imaging of cells depleted of TAAC3 and ROD1 was performed using a DeltaVisionElite system fluorescent microscope (GE Healthcare) equipped with a CoolSNAP HQ2 camera (Photometrics), 40× oil immersion objective.

Cells were seeded in an 8‐well ibidi dish (Ibidi) the day before filming; the media was changed to Leibovitz's L‐15 (Life Technologies) immediately before the filming. YFP (10% power, exposure 0.2) and mCherry (5% power, 0.05 exposure) were recorded at 7 min intervals, and data were analyzed using SoftWoRx (GE Healthcare). The time from nuclear envelope breakdown (NEBD) to anaphase was measured in single cells from independent experiments. Further analysis carried out with GraphPad Prism 9 (GraphPad Software, San Diego, California, USA).

### Cell growth assays

The Sulforhodamine B (SRB) proliferation assay was used to assess *in vitro* cytotoxicity of the compounds (Vichai & Kirtikara, 2006). Cells were treated with sublethal drug concentrations (LD20) for 12 days from the day of seeding. If necessary, transient siRNA transfection was performed 3 days before drug treatment. After 12 days, medium was removed from the wells and cells were washed once with PBS and fixed in prechilled 10% trichloroacetic acid (TCA) (Sigma‐Aldrich) for 30 min at 4°C. Cells were subsequently washed two times with double‐distilled water and stained with 0.4% SRB (1% acetic acid for 20 min at room temperature protected from light). SRB was then removed, and cells were washed four times in 1% acetic acid. After complete light‐protected drying, SRB was dissolved by addition of 10 mM Tris pH 8 and gentle shaking at room temperature for 2 h. Hundred microliters of the solution were then transferred to a 96‐well plate and absorbance at A_510_ was read with plate reader. Percentage of cell growth inhibition was calculated in relation to a mock‐treated control.

The IncuCyte^®^ live‐cell analysis system was additionally used to real‐time track the growth of the cells subjected to the different treatments.

### Live imaging of DT‐061‐treated cells

HeLa or H358 parental cells were seeded in 8‐well dish (Ibidi) and incubated for a minimum of 16 h with the fusion construct CellLight™ Golgi‐GFP or CellLight™ ER‐RFP, BacMam 2.0 (Invitrogen™). Right before imaging, media was exchanged to Leibovitz’s L‐15 (Life Technologies). DT‐061 was added to the ongoing experiment for a final concentration of 20 µM.

Filming was performed on an LSM 880 Airyscan Confocal attached to an inverted stand Zeiss AxioObserver.Z1 (Carl Zeiss), equipped with an Incubation box XL Multi S1 set to 37°C, and sample was mounted on a C‐Apochromat ×40/1.2W objective. Time‐lapse series of 10 min, 6–7 z‐stacks, 1AU pinhole aperture. Image acquisition was performed with ZEN 2.1 software (Carl Zeiss).

A conventional detector was used to acquire the GFP or RFP signal (laser line Argon 25 mW and DPSS 10 mW, respectively) while the signal from the drug DT‐061 was acquired through the laser line Diode 30 mW using a highly sensitive multiarray 32PMT GaAsP detector.

The same was performed for iHAP1 for a final concentration of 2 µM as a negative control. Stills and videos were further processed using the software Fiji (Schindelin *et al*, 2012).

### Lipidomics

A TNF‐sensitive subclone (MCF7‐S1) of the MCF7 human ductal breast carcinoma cell line (Jaattela *et al*, 1995) (hereafter referred to as MCF7) was cultured in RPMI medium (Gibco) supplemented with 6% FCS and in a humidified incubator set to 37°C and 5% CO_2_. Approximately, 1 million MCF7 cells cultured in a 6‐well dish were treated with 15 µM DT‐061, 1.5 µM iHAP1, 3 µg/ml Brefeldin A or vehicle (DMSO) for 30 min. We then added 10 µM sphingosine (Avanti Polar Lipids, Alabama, USA) or vehicle (DMSO) to the medium and cultured them for additional 4 h. The cells were then washed five times with ice‐cold 155 mM ammonium bicarbonate and harvested into 1 ml 155 mM ammonium bicarbonate by scraping. Two hundred microliters of the cell suspensions were then subjected to lipid extraction and quantitative mass spectrometry‐based lipidomics as described previously (Nielsen *et al*, 2020). The criteria used to identify ceramide, sphingomyelin, hexosylceramide, GM3 and PC are listed in Appendix Fig S11. The internal standards used for quantification of lipids are listed in Appendix Fig S12.

### Experimental design and statistical analysis

No statistical method was used to predetermine sample size, and we have not used randomization or blinding procedures. We have only excluded experiments from analysis where the controls did not work.

Statistical analysis and graphs were generated in GraphPad Prism 8.0. The data points were tested for normality using Shapiro–Wilk test. Accordingly, statistical significance was determined by unpaired Student's *t*‐test or Mann–Whitney *U* test. *F* test was used to compare variances and for conditions that did not have equal variances; parametric tests with Welch's correction was used. Details of the statistical significance and *n* values for each condition can be found in the figures and figure legends.

## Author contributions

**Gianmatteo Vit:** Conceptualization; Formal analysis; Supervision; Funding acquisition; Validation; Investigation; Visualization; Methodology; Writing—original draft; Writing—review & editing. **Joana Duro:** Conceptualization; Data curation; Formal analysis; Validation; Investigation; Visualization; Methodology; Writing—original draft; Writing—review & editing. **Girish Rajendraprasad:** Conceptualization; Data curation; Formal analysis; Validation; Investigation; Visualization; Methodology; Writing—original draft; Writing—review & editing. **Emil P T Hertz:** Conceptualization; Data curation; Formal analysis; Investigation; Visualization; Methodology; Writing—review & editing. **Lay Katrine Kauffeldt Holland:** Conceptualization; Data curation; Formal analysis; Validation; Investigation; Visualization; Methodology; Writing—review & editing. **Melanie Bianca Weisser:** Data curation; Formal analysis; Validation; Investigation; Visualization; Methodology; Writing—review & editing. **Brennan C McEwan:** Formal analysis; Investigation; Visualization; Methodology; Writing—review & editing. **Blanca Lopez‐Mendez:** Formal analysis; Investigation; Visualization; Methodology; Writing—review & editing. **Paula Sotelo‐Parilla:** Formal analysis; Investigation; Visualization; Methodology; Writing—review & editing. **A Arockia Jeyaprakash:** Supervision; Funding acquisition; Investigation; Methodology; Writing—review & editing. **Guillermo Montoya:** Formal analysis; Supervision; Funding acquisition; Investigation; Visualization; Writing—review & editing. **Niels Mailand:** Formal analysis; Supervision; Funding acquisition; Visualization; Writing—review & editing. **Kenji Maeda:** Formal analysis; Supervision; Funding acquisition; Investigation; Visualization; Writing—review & editing. **Arminja Kettenbach:** Formal analysis; Supervision; Funding acquisition; Investigation; Visualization; Writing—review & editing. **Marin Barisic:** Formal analysis; Supervision; Funding acquisition; Investigation; Visualization; Writing—review & editing. **Jakob Nilsson:** Conceptualization; Formal analysis; Supervision; Funding acquisition; Investigation; Visualization; Methodology; Writing—original draft; Writing—review & editing.

## Disclosure and competing interests statement

JN has been on the advisory board for Orion Pharma during the project. GM is a co‐founder and board member of Twelve Bio. The rest of the authors declare that they have no conflict of interest.

## Supporting information



Appendix

Expanded View Figures PDF

Dataset EV1

Dataset EV2

Dataset EV3

Movie EV1

Movie EV2

Movie EV3

Movie EV4

Movie EV5

Movie EV6

Movie EV7

Movie EV8

Source Data for Figure 2

Source Data for Figure 3

Source Data for Figure 4

Source Data for Figure 5

Source Data for Figure 6

Source Data for Figure 7

Source Data for Figure 8

## Data Availability

The mass spectrometry data have been deposited to ProteomeXchange PXD026666 (http://proteomecentral.proteomexchange.org/cgi/GetDataset?ID=PXD026666).
