# Supplementary material for: Chemogenetic profiling reveals PP2A‐independent cytotoxicity of proposed PP2A activators iHAP1 and DT‐061
Source: EMBO J. 2022 Jun 13;41(14):e110611. doi: 10.15252/embj.2022110611 (PMC9289710; doi:10.15252/embj.2022110611)
Supplement: Supplementary file 1 — Appendix [file EMBJ-41-e110611-s020.pdf]

Appendix

Chemogenetic profiling reveals PP2A-independent cytotoxicity of proposed PP2A activators iHAP1 and DT-061

Table of contents

Appendix Figure S1. Purity of PP2A holoenzyme preparation and mass photometry measurements.

Appendix Figure S2. Calorimetric titrations of PPP2R1A with perphenazine, DT-061 and iHAP1.

Appendix Figure S3. NMR analysis of PPP2R1A interaction with Perphenazine.

Appendix Figure S4. NMR analysis of PPP2R1A interaction with iHAP1 and DT-061.

Appendix Figure S5. Magnification of NMR analysis of PPP2R1A interaction with iHAP1.

Appendix Figure S6. Size-exclusion chromatography of HeLa total cell extract.

Appendix Figure S7. Size-exclusion chromatography of H358 total cell extract.

Appendix Figure S8. Analysis of the Cryo-EM structure of DT-061 bound to PP2A-B56α

Appendix Figure S9. iHAP1 has no effect on Golgi and ER morphology in HeLa cells.

Appendix Figure S10. DT-061 impairs ER morphology in H358 cells.

Appendix Figure S11. Precursor ions and fragment ions used for lipid identification.

Appendix Figure S12. Internal lipid standards

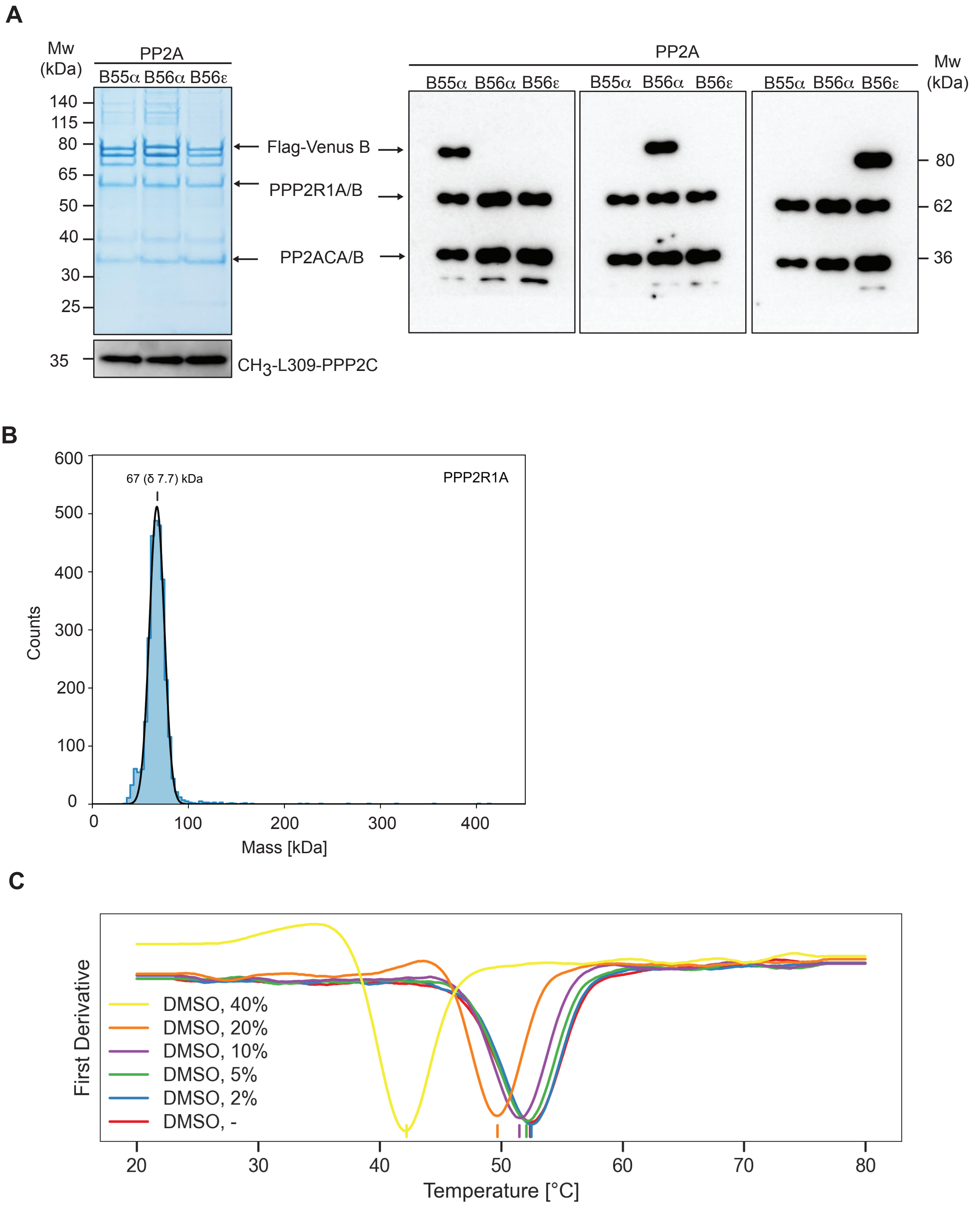

Appendix Fig. S1

Appendix Figure S1. **Purity of PP2A holoenzyme preparation and mass photometry measurements.**

A) Analysis of the purity of PP2A holoenzyme preparations by colloidal staining and Western blotting. Methylation of the C-tail of PPP2C was confirmed using an antibody against CH3-L309. Samples were analyzed by mass spectrometry which confirmed that no other protein phosphatases were present in the preparations. Two enzymatic preparations were made. Shown are a representative colloidal staining and Western blot. B) MP mass distribution of PPP2R1A in NMR buffer (PBS). The mass photometry measurement of PPP2R1A shows a major peak with molecular weight of 67 kDa in agreement with the molecular weight of the monomeric PPP2R1A. Three technical replicates of the experiment were performed. C) First derivative of the integrated F350/F330 nm fluorescence ratio thermal unfolding curves color coded as increasing percentages of DMSO in ITC buffer. Tm values of each sample are indicated by vertical lines.

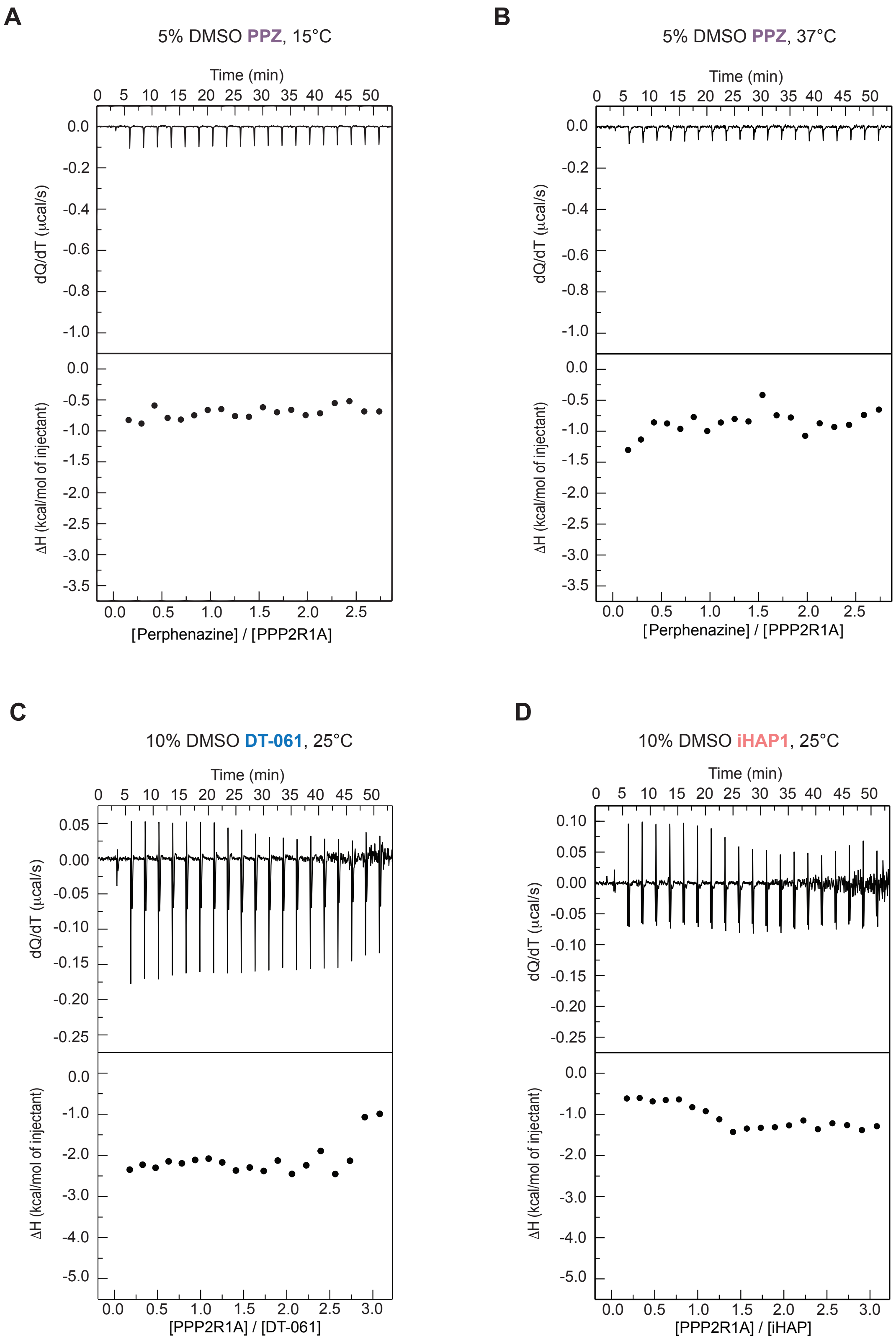

Appendix Fig. S2

Appendix Figure S2. Calorimetric titrations of PPP2R1A with perphenazine, DT-061 and iHAP1.

A-B) Thermogram (top panel) for the calorimetric titration of perphenazine (400 µM, in syringe) and corresponding ligand-normalized integrated heats (bottom panel) for PPP2R1A (30 µM, in calorimetric cell) at 15°C (left) and 37°C (right). C-D) Same as in A-B) but with PPP2R1A (300 µM, in syringe) and DT-061 (20 µM, in calorimetric cell) and iHAP1 (20 µM, in calorimetric cell) at 25 °C. Experiments were performed in 5% (A-B) and 10% DMSO (C-D) in 100 mM sodium phosphate pH 7.5, 150 mM NaCl, 0.5 mM TCEP. A-D) Shown are the representative results of at least two independent repeats.

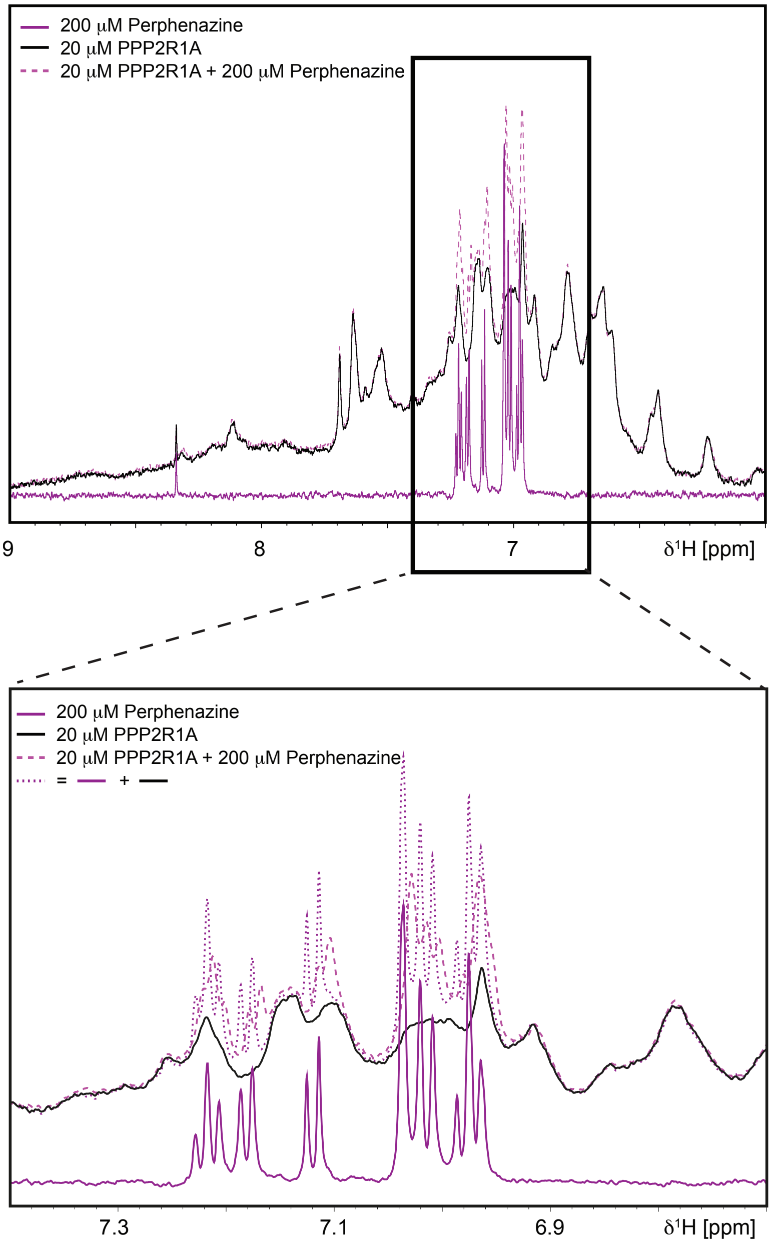

Appendix Figure S3. NMR analysis of PPP2R1A interaction with perphenazine.

Aromatic- and Amide-proton region of the 1D-1H-NMR spectra of 20 µM PPP2R1A alone (black) and in presence of 20 µM perphenazine (violet dotted) or 20 µM perphenazine alone (violet), respectively. Each sample were analyzed in two scans.

Appendix Fig. S3

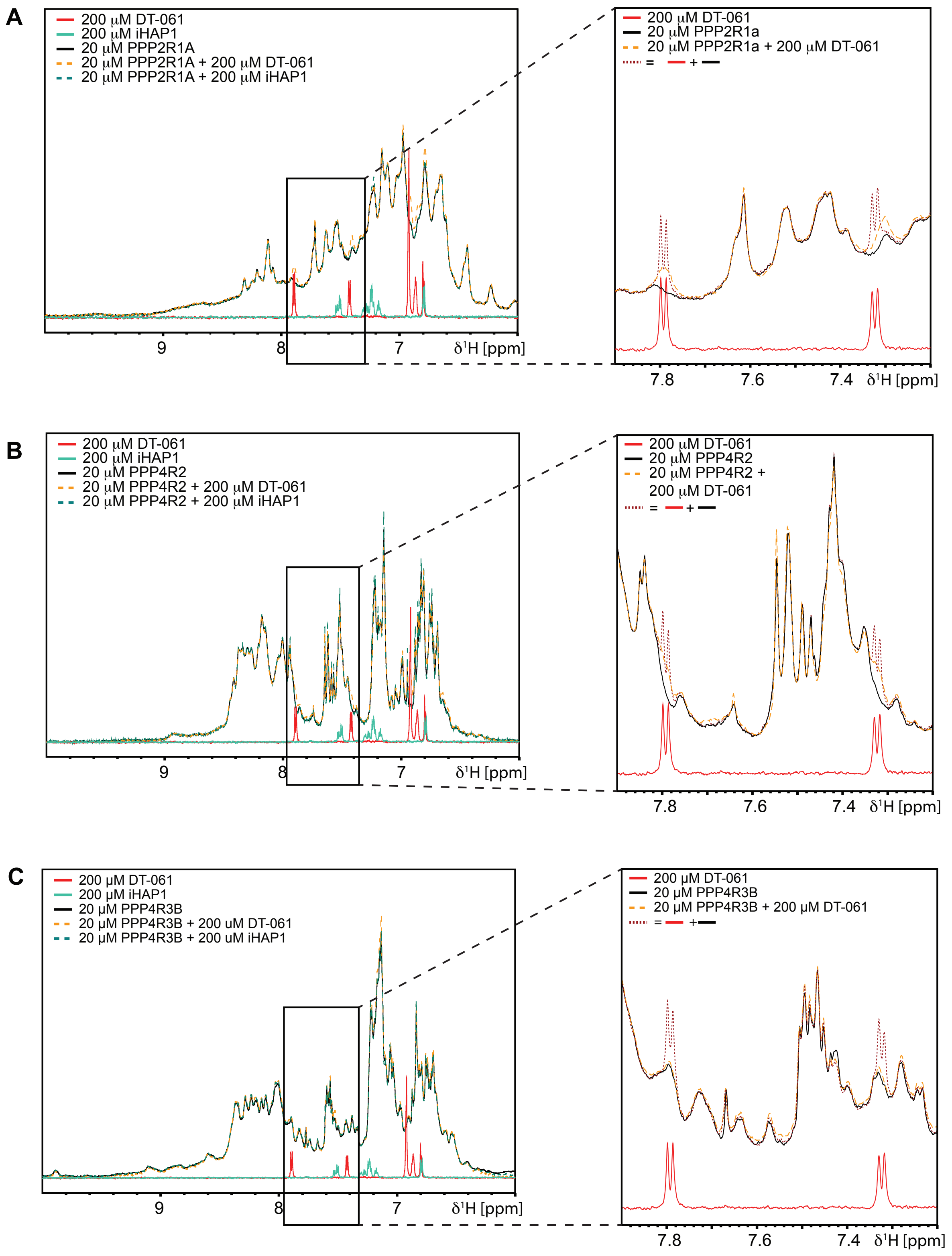

Appendix Fig. S4

Appendix Figure S4. NMR analysis of PPP2R1A interaction with iHAP1 and DT-061.

1D-1H NMR fingerprint spectra of the amide and aromatic region of PPP2R1A (A), PPP4R2 (B) and PPP4R3B (C). The NMR spectrum of the respective free protein is shown in black, free iHAP1 is shown in sky-blue, free DT-061 in red and the protein-compound mixtures are shown as dashed lines (cyan for iHAP and orange for DT-061). (A-C) Magnifications of the spectral region with signals of the compounds are shown on the right where the mathematical sum of the spectra of free compound and free protein are shown as dotted lines (this spectrum would be the expected outcome if the two molecules are completely independent in solution, without any unspecific interactions). Drug and enzyme concentrations used in the experiment are stated in the figure. Each sample were analyzed in two scans.

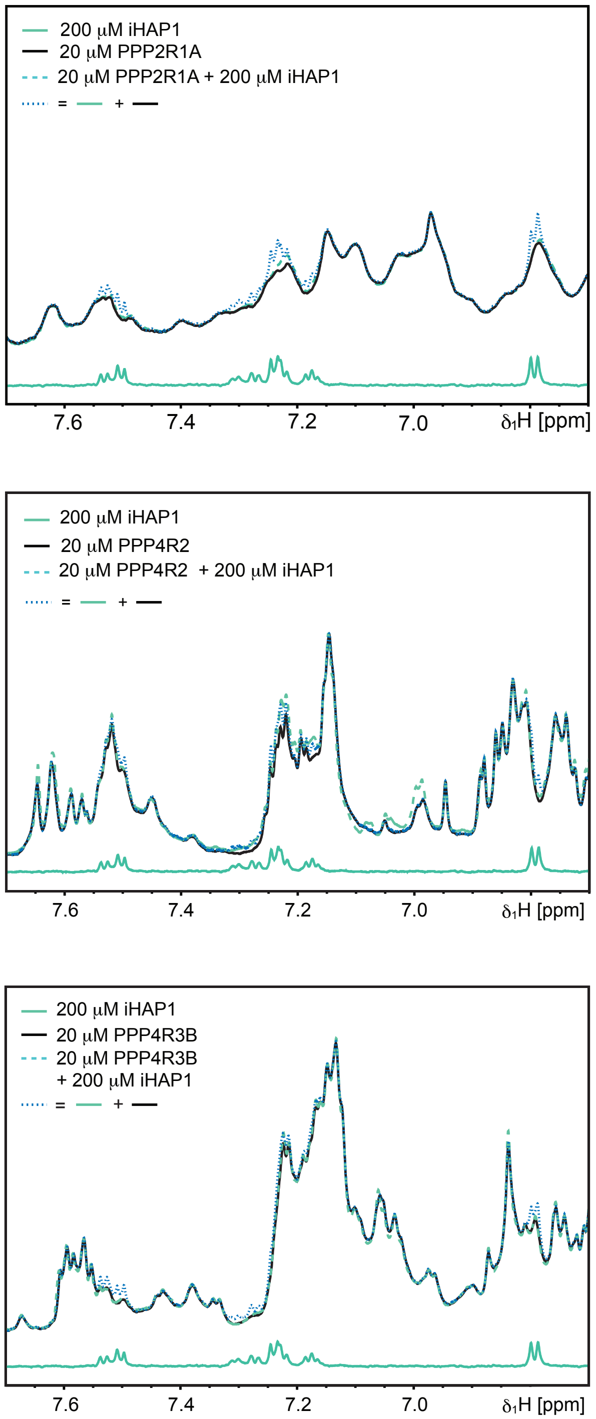

Appendix Figure S5. Magnification of NMR analysis of PPP2R1A interaction with iHAP1.

A-C) Magnifications of the spectral region with signals of the compound iHAP1 related to Appendix Fig. S4. Drug and enzyme concentrations used in the experiment are stated in the figure. Each sample were analyzed in two scans.

Appendix Fig. S5

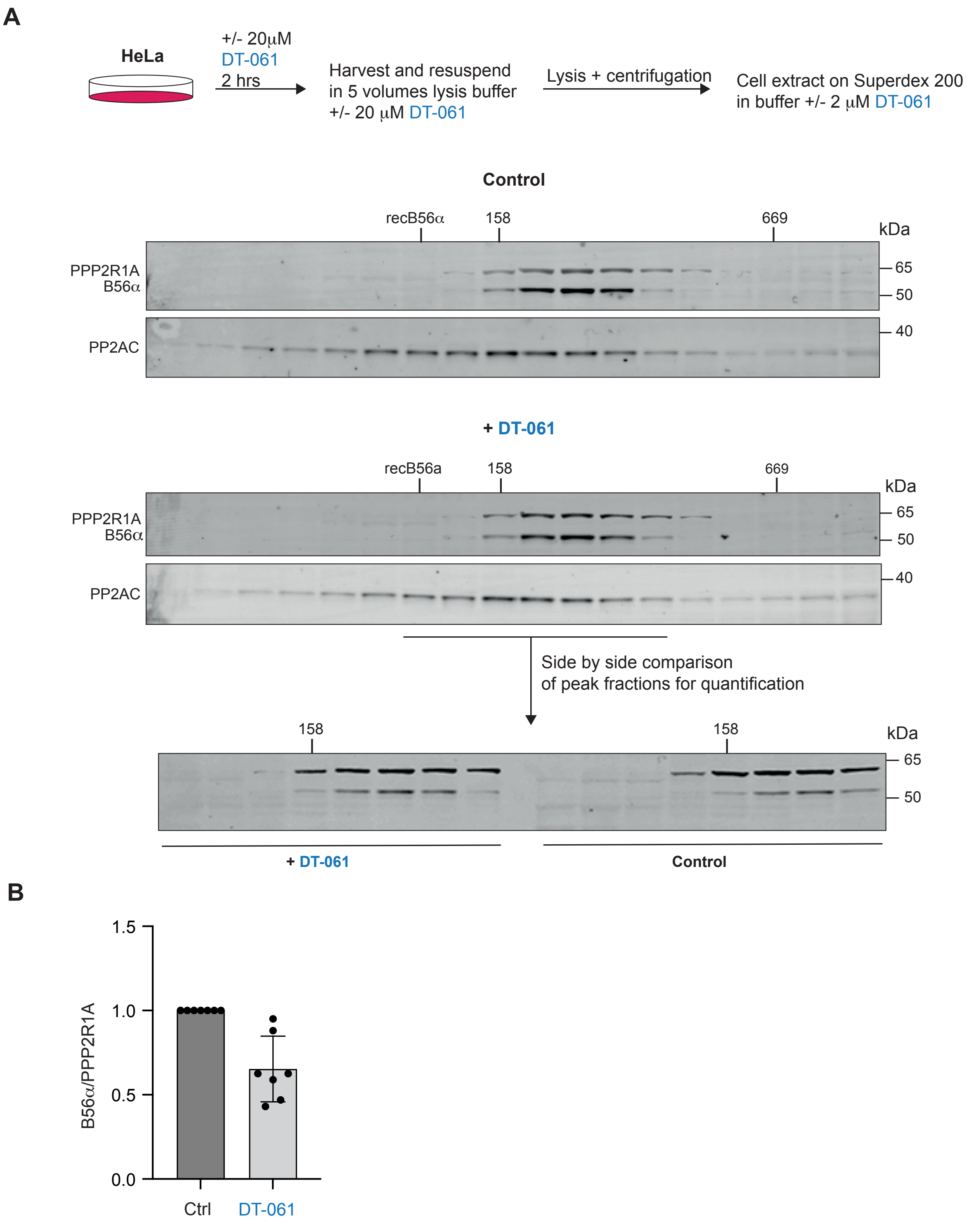

Appendix Figure S6. Size-exclusion chromatography of HeLa total cell extract.

A-B) A total cell extract was prepared from HeLa cells either untreated or treated for 2 hours with 20 µM DT-061. For the DT-061 treated sample the lysis buffer contained 20 µM DT-061. The complexes in the cell extracts were separated on a Superdex200 column with the buffer containing 2 µM DT-061 for the DT-061 treated sample. The migration of molecular weight markers or recombinant untagged B56α is indicated on top. The fractions were analyzed by quantitative Licor Western blot for PPP2R1A, B56α and PP2AC. For a quantitative comparison the peak fractions were run on the same gel and the ratio of B56α to PPP2R1A calculated for the peak fractions. Representative of 3 biological replicates. B) Mean and individual measurements shown as well as SD.

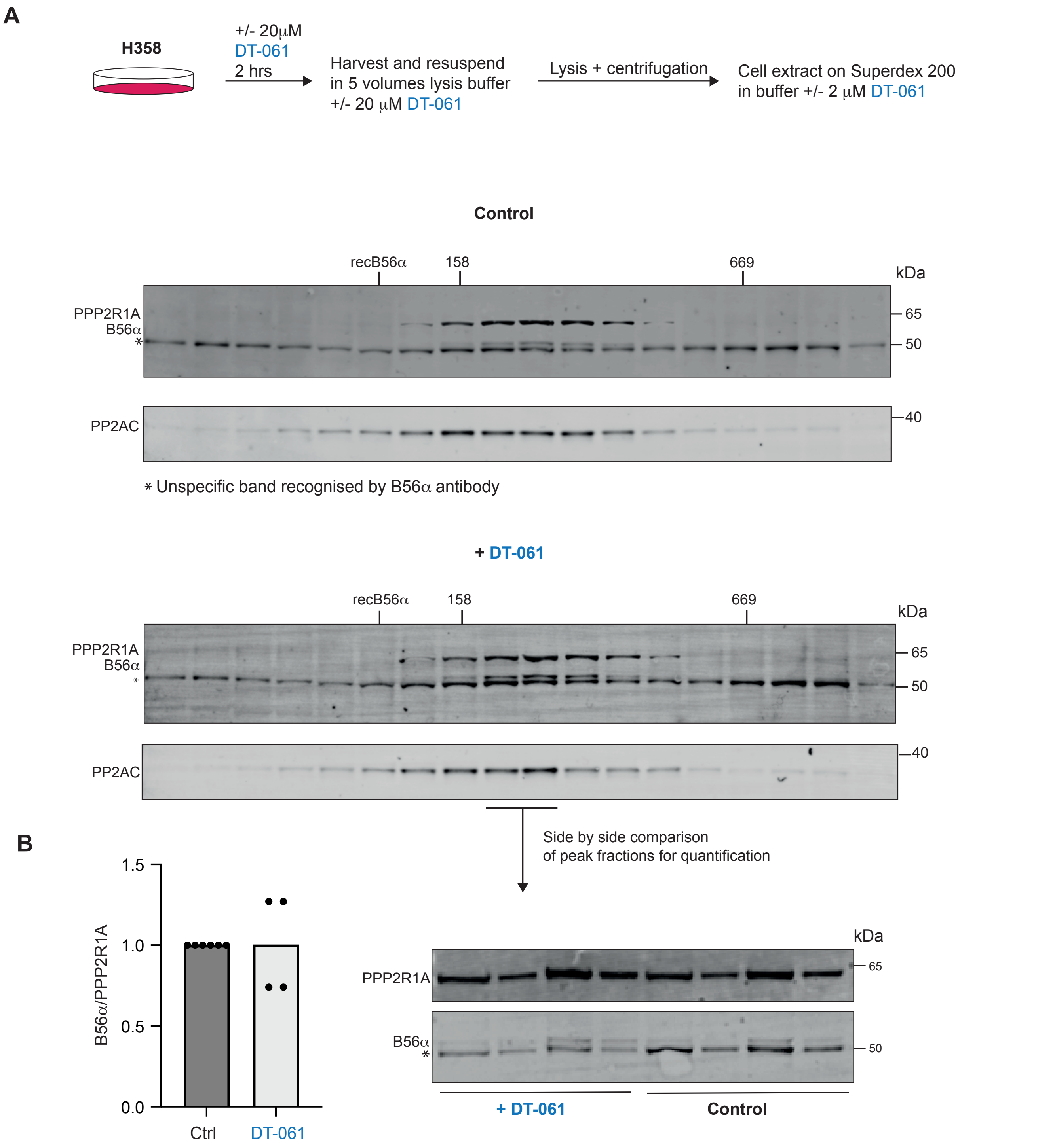

Appendix Figure S7. Size-exclusion chromatography of H358 total cell extract.

A) A total cell extract was prepared from H358 cells either untreated or treated for 2 hours with 20 μM DT-061. For the DT-061 treated sample the lysis buffer contained 20 μM DT-061. The complexes in the cell extracts were separated on a Superdex200 column with the buffer containing 2 μM DT-061 for the DT-061 treated sample. The migration of molecular weight markers or recombinant untagged B56α is indicated on top. The fractions were analyzed by quantitative Licor Western blot for PPP2R1A, B56α and PP2AC. For a quantitative comparison the peak fractions were run on the same gel and the ratio of B56α to PPP2R1A calculated for the peak fractions. Representative of 2 biological replicates. B) Mean and individual data points shown from two biological replicates.

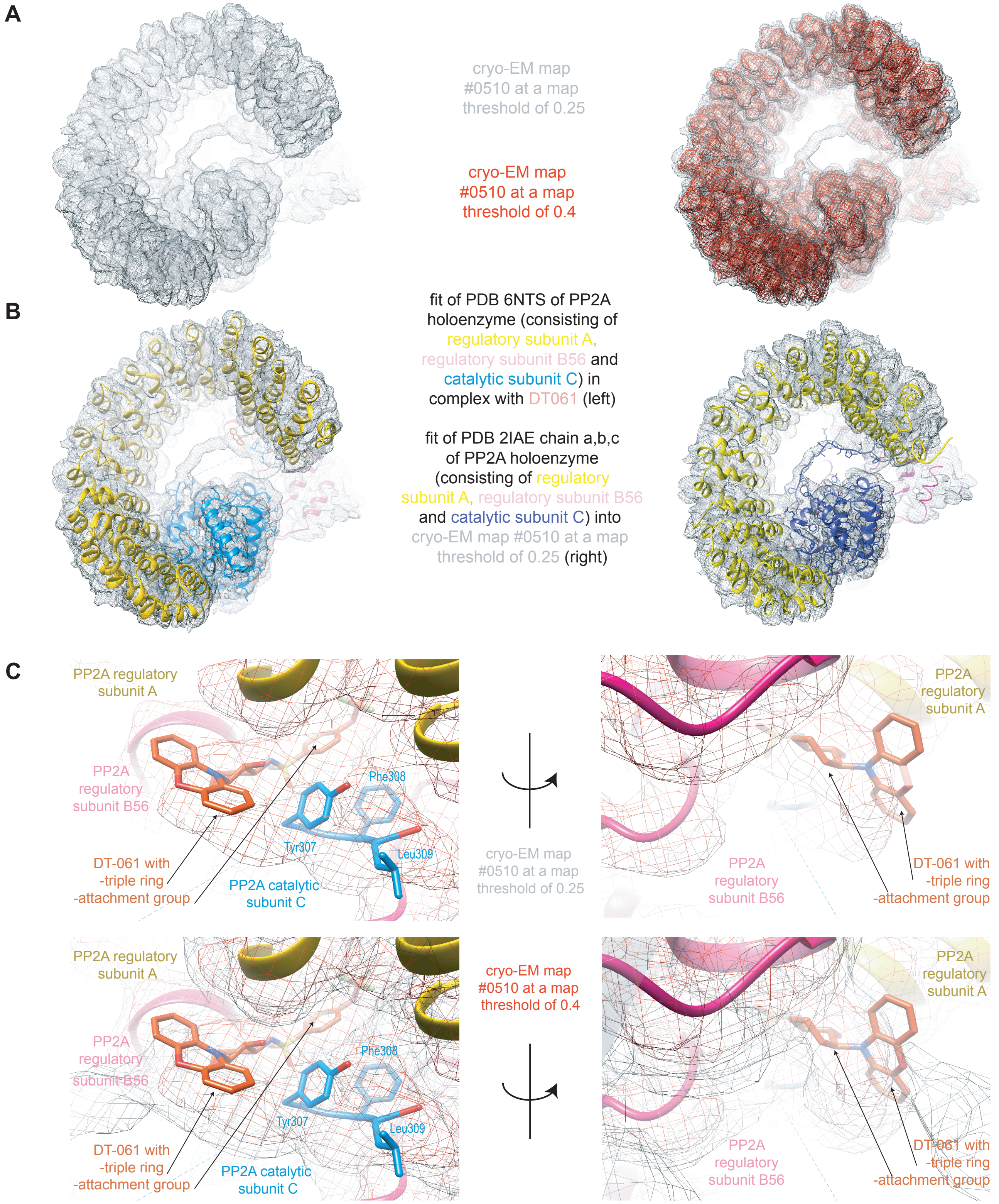

Appendix Fig. S8

Appendix Figure S8. Analysis of the Cryo-EM structure of DT-061 bound to PP2A-B56.

A) Views of EMD-0510 map at two different density threshold levels. Lower threshold levels (0.25, grey, left panel) reveal a continuous stretch of extra density which spans the center of the horseshoe-shaped holoenzyme complex. This density is not visible at higher thresholds (0.4, red, right panel) indicating that it corresponds to a part of the holoenzyme that is structurally more flexible than the rest. B) The left-hand panel shows a fit of PDB 6NTS into the EMD-0510 map, comprising the regulatory A subunit (yellow), the catalytic C subunit (blue) and regulatory B56 subunit (pink). The fit shows that the low threshold extra density connects the main body of the PP2A/C subunit (modelled residues 1-294, blue) with the three most C-terminal residues of the C subunit (residues 307-309, blue) that are in contact with the regulatory A subunit and in the immediate vicinity of where the DT-061 ligand has been placed (red, left panel). Residues 295-306 are absent from this model of the C subunit. In the right-hand panel, a complete model of the C subunit that contains residues 295-306 (from PDB model 2IAE chain c, shown dark blue, right panel) has been superimposed on the C subunit of PDB 6NTS and map EMD-0510 (at threshold of 0.25). The superposition shows that the presence of residues 295-306 could also explain the stretch of extra density in the EMD-0510 map. The presence of these residues would promote a steric clash with the suggested position of DT-061. C) Close-up view of the suggested DT-061 ligand fit into EMD-0510 map. The two views (left- and right-hand panel) and the two different map thresholds (grey 0.25 and red 0.4, bottom panels) show that the ligand's attachment group is embedded quite well into the cryo-EM map, but the tricyclic heterocyclic moiety fit appears suboptimal even at lower map thresholds.

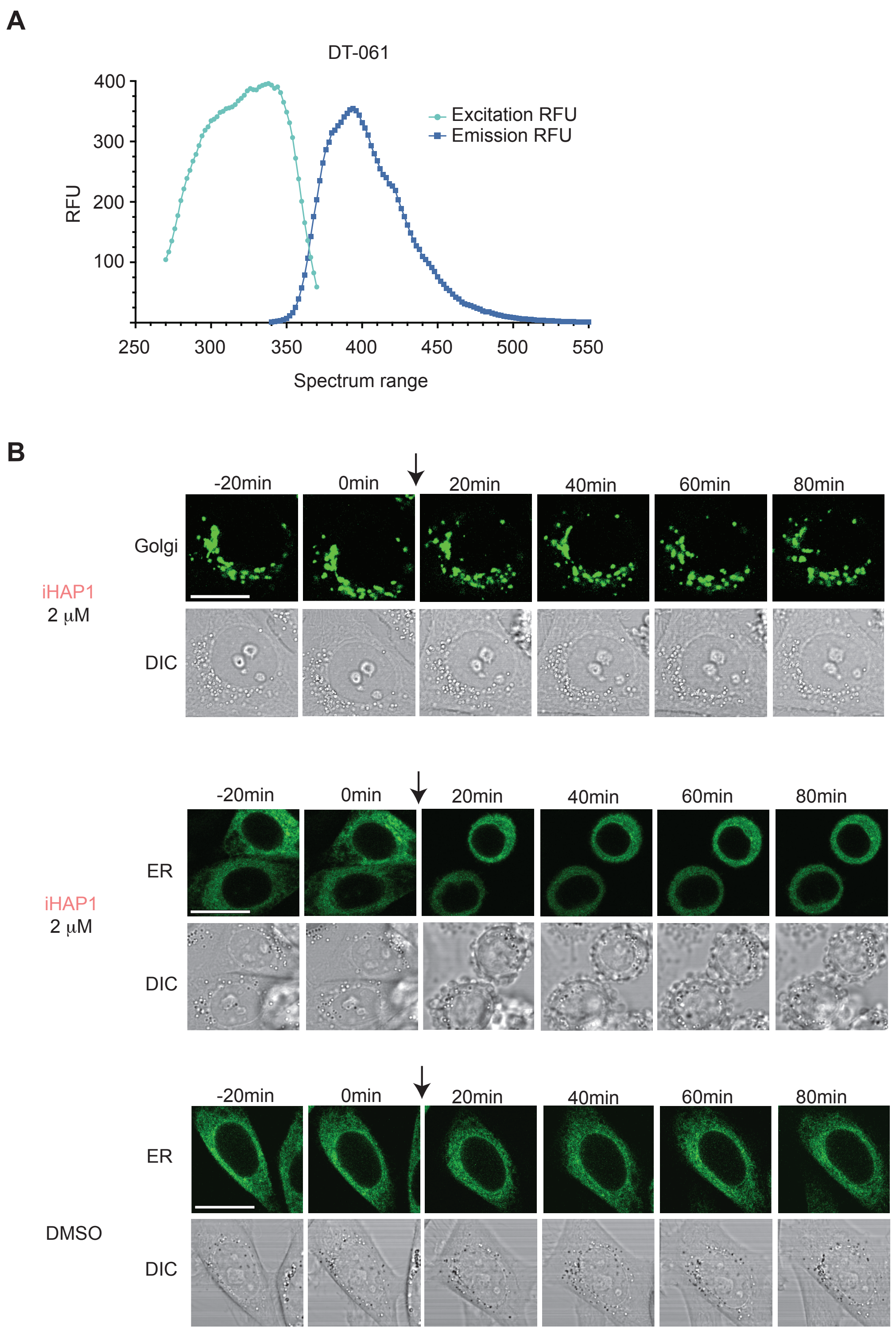

Appendix Figure S9. iHAP1 has no effect on Golgi and ER morphology in HeLa cells.

A) Excitation/emission spectrum for DT-061. B) Time-lapse imaging of HeLa cells expressing a marker staining the Golgi (upper panel) and ER (middle and low panels). Cells were treated with 2 µM iHAP1 or DMSO (Ctrl). Experiments were repeated at least two independent times and stills show a representative cell for each treatment condition. 234 cells were analyzed for the ER stained cells treated with iHAP1 and no cells showed translocation of the ER marker. Arrow indicates the addition of the drug. Scale bar indicates 20µm.

Appendix Fig. S9

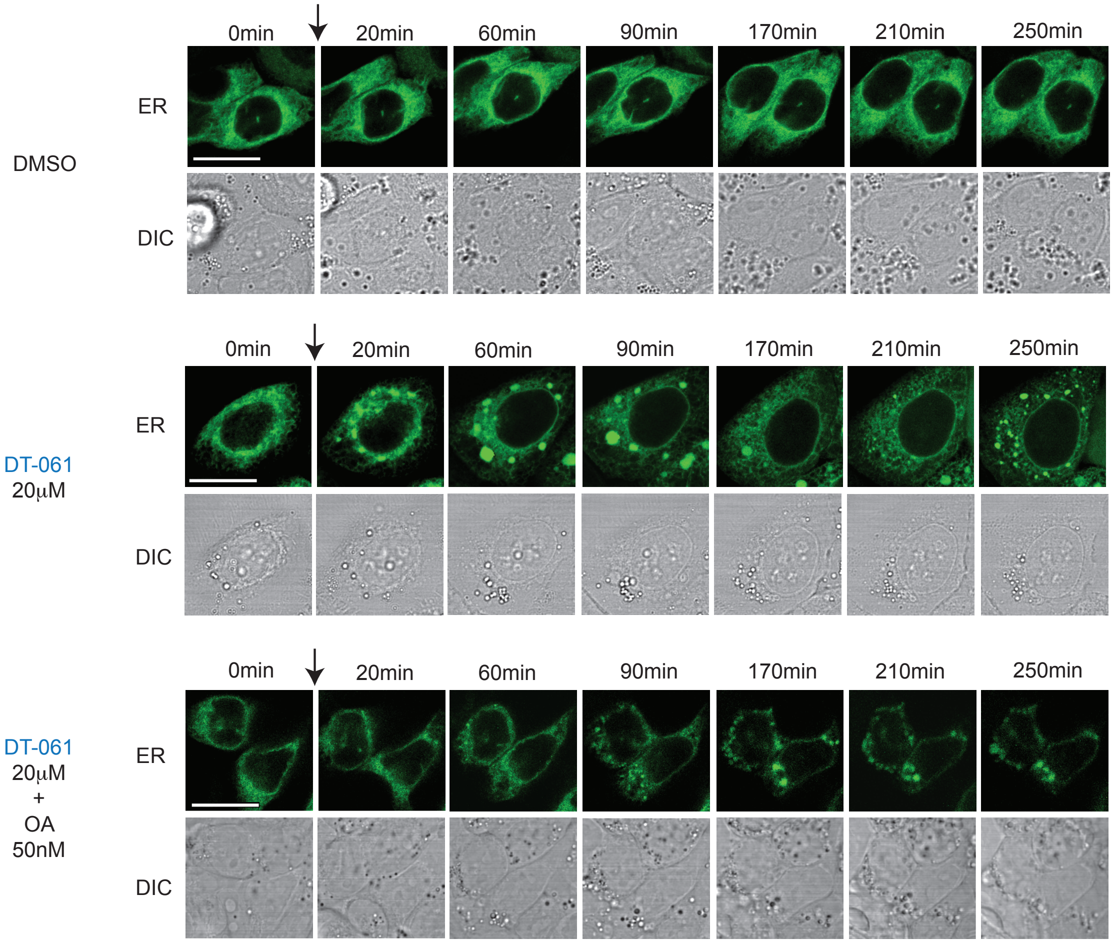

Appendix Figure S10. DT-061 impairs ER morphology in H358 cells.

Time-lapse imaging of H358 cells expressing a marker staining ER. Cells were mock-treated with DMSO (Ctrl, upper panel), 20 μM DT-061 alone (middle panel) or 20 μM DT-061 and 50 nM OA (low panel). Example of phenotype where the ER marker did not translocate. Experiments were repeated at least twice and stills show a representative cell for each treatment condition. Arrow indicates the addition of the drug. Scale bar indicates 20μm.

Appendix Fig. S10

**Table: Precursor ions and fragment ions used for lipid identification**					
**Lipid class**	**Mode**	**MS1: Precursor ion**	**MS2: Fragment ion**	**MS2: *m/z***	**MS2: Species specifics**
PC and SM	POS	$[M+H]^+$	$[Phosphorylcholine + H]^+$	184.0733	All
Cer and HexCer	POS	$[M+H]^+$	$[LCB + H – H_2O]^+$	*	All
		$[M+H]^+$	$[LCB + H – 2H_2O]^+$	*	All
GM3	NEG	$[M–H]^-$	$[NeuAc – H]^-$	290.0864	All

* Depends on species.

Appendix Fig. S11

Table: Internal lipid standards				
**Lipid class**	**Sum formula**	**Source**	**ID**	**Amount added (pmol)**
PC	PC 12:0/12:0	Avanti Polar Lipids	850335	20
Cer	Cer 18:1;2/12:0;0	Avanti Polar Lipids	860512	16
HexCer	HexCer 18:1;2/12:0;0	Avanti Polar Lipids	860543	20
GM3	GM3 18:1;2/18:0;0-D3	Larodan	71-1107	26
SM	SM 18:1;2/12:0;0	Avanti Polar Lipids	860583	13.62

Appendix Fig. S12
