# Supplementary material for: Chemogenetic profiling reveals PP2A‐independent cytotoxicity of proposed PP2A activators iHAP1 and DT‐061
Source: EMBO J. 2022 Jun 13;41(14):e110611. doi: 10.15252/embj.2022110611 (PMC9289710; doi:10.15252/embj.2022110611)
Supplement: Supplementary file 2 — Expanded View Figures PDF [file EMBJ-41-e110611-s012.pdf]

Expanded View Figures

Figure EV1. Perphenazine, iHAP1 and DT-061 do not activate PP2A complexes *in vitro*.

A Titration experiment of different PP2A holoenzyme preparations to determine appropriate concentration to use in enzymatic assays. Experiment was performed twice using the concentrations of enzyme stated in the figure. Shown is one representative experiment.

B–E *In vitro* dephosphorylation assays using model phosphopeptides and purified PP2A holoenzymes. The phosphopeptides were incubated with specific PP2A holoenzymes together with PPZ, iHAP1 or DT-061 at the indicated concentrations. The reaction was stopped after 15 min, and the amount of phosphate released was measured. In (E) substrates are BRCA2 WT: P(pS)QKAEITELSTILEESGSQW and BRCA2 2A: P(pS)QKAEITEASTALEESGSQW. Mean and SD from three technical replicates of one out of three independent experiments is shown. Experiment in (E) is representative of two independent experiments.

F Injection of PPP2R1A (300 μM) into the ITC buffer (5% DMSO) used for the DT-061, iHAP experiments. The experiments were performed at 25°C. No significant effects due to dilution of protein (control experiment for Fig 1B)

© 2022 The Authors

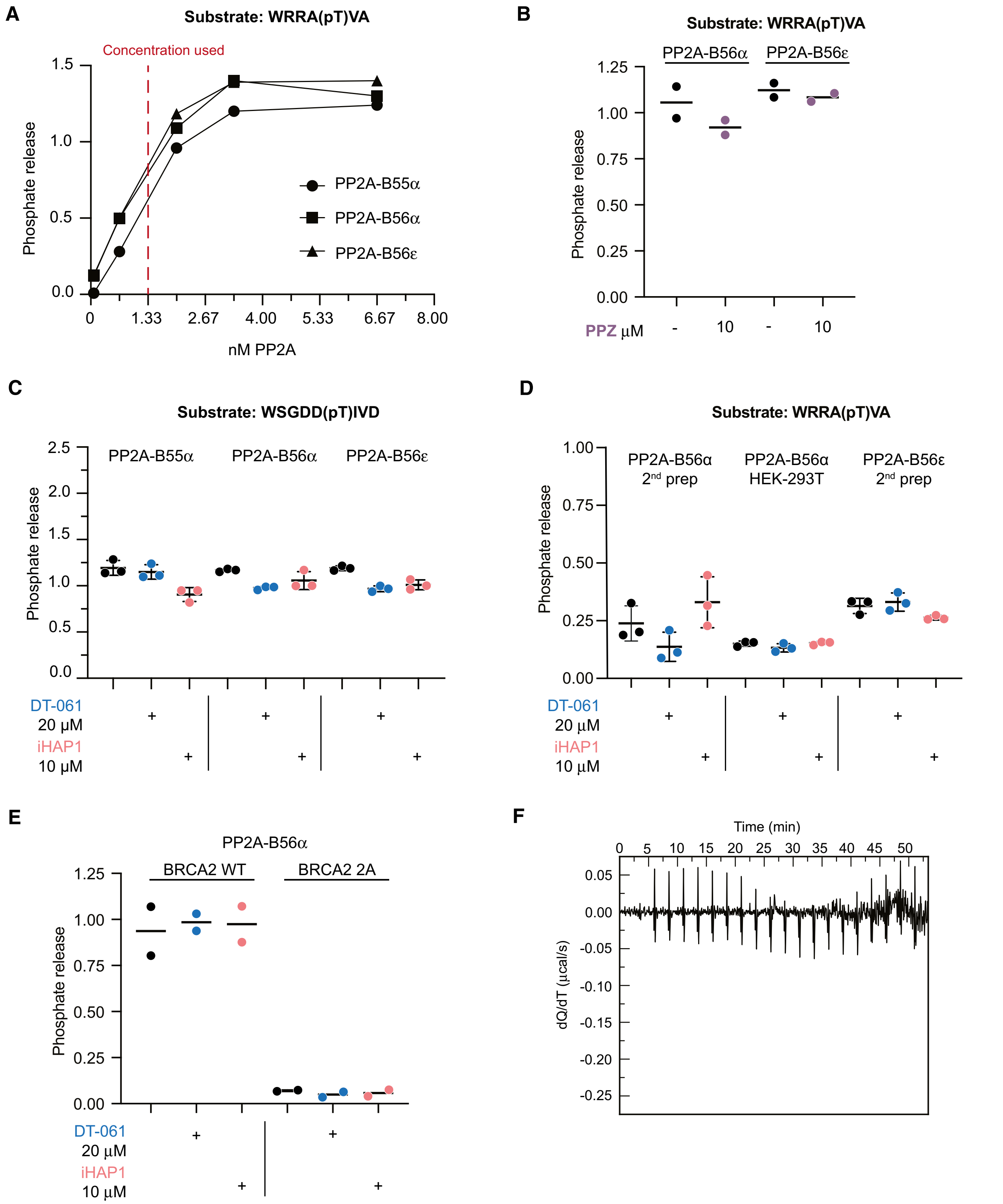
A
Substrate: WRRA(pT)VA
Concentration used
Phosphate release
1.5
1.0
0.5
0.0
0
1.33
2.67
4.00
5.33
6.67
8.00
nM PP2A
PP2A-B55α
PP2A-B56α
PP2A-B56ε
B
Substrate: WRRA(pT)VA
PP2A-B56α
PP2A-B56ε
1.25
1.00
0.75
0.50
0.25
0.00
Phosphate release
PPZ μM
-
10
-
10
C
Substrate: WSGDD(pT)IVD
PP2A-B55α
PP2A-B56α
PP2A-B56ε
2.5
2.0
1.5
1.0
0.5
0.0
Phosphate release
DT-061
20 μM
iHAP1
10 μM
D
Substrate: WRRA(pT)VA
PP2A-B56α
2nd prep
PP2A-B56α
HEK-293T
PP2A-B56ε
2nd prep
1.00
0.75
0.50
0.25
0.00
Phosphate release
DT-061
20 μM
iHAP1
10 μM
E
PP2A-B56α
BRCA2 WT
BRCA2 2A
1.25
1.00
0.75
0.50
0.25
0.00
Phosphate release
DT-061
20 μM
iHAP1
10 μM
F
Time (min)
0
5
10
15
20
25
30
35
40
45
50
0.05
0.00
-0.05
-0.10
-0.15
-0.20
-0.25
dQ/dT (μcal/s)

Figure EV1.

Figure EV2. ***In vivo*** **and structural analysis of drug activity on cells.**

A CRISPR-Cas9-inducible HeLa cell lines for PP2A regulatory subunits. Western blotting shows the removal of the indicated B56 subunits 3 and 12 days after addition of doxycycline, respectively. Actin was used as loading control. At least three biological replicates of the experiment were performed.

B Incucyte growth assay using a HeLa cell line stably expressing inducible YFP-B56α. Induced cells were treated with Doxycycline for 12 h before imaging. Cell was treated with 20 μM DT-061 or DMSO (Ctrl). Experiment was repeated in three times independently of each other with each experiment performed in technical triplicate. A representative independent experiment is shown with mean and SD.

C A total cell extract was prepared from HeLa cells stably expressing doxycycline-inducible YFP-B56α. Cells were either untreated or expression induced for 24 h with Doxycycline and the complexes in the cell extracts separated on a Superdex200 column. The migration of molecular weight markers or recombinant untagged B56α is indicated on top. The fractions were analyzed by quantitative Licor western blot for PP2A/A, B56α and PP2A/C. The ratio of B56α or Venus-B56α to PP2A/A in the peak fractions is shown below. PP2AC levels co-migrating with PPP2R1A increased 2–3-fold in the sample where YFP-B56α was induced. Experiment was performed once.

D (i) The superposition of the crystallographic electron densities maps of PDB 2IAE chain a,b,c (green) and PDB 3FGA (light pink) with cryo-EM map EMD-0510 (gray) at a map threshold of 0.25 shows that in all three maps, density is protruding into the center of the horseshoe-shaped PP2A holoenzyme at the same position (closeup, right). The fits of all three PP2A structures—PDB 6NTS (ii), PDB 3FGA (iii) and PDB 2IAE, (chain a,b,c) (iv) —into their respective densities show that in all three cases, this area of density was attributed to the C-terminal tail of the PP2A catalytic subunit. In the case of PDB 6NTS, the overall map quality and local resolution in that area reveals less easily interpretable features than in the case of PDBs 3FGA/2IAE. Only the last three C-terminal tail residues (307-9) were built, and the DT061 ligand (red) was modeled right next to them. This leaves most of the visible extra density (asterisk) uninterpreted (ii), in a position where in the higher resolution models of PP2A/C (3FGA/2IAE) further residues of the PP2A/C C-terminus have been placed.

 © 2022 The Authors

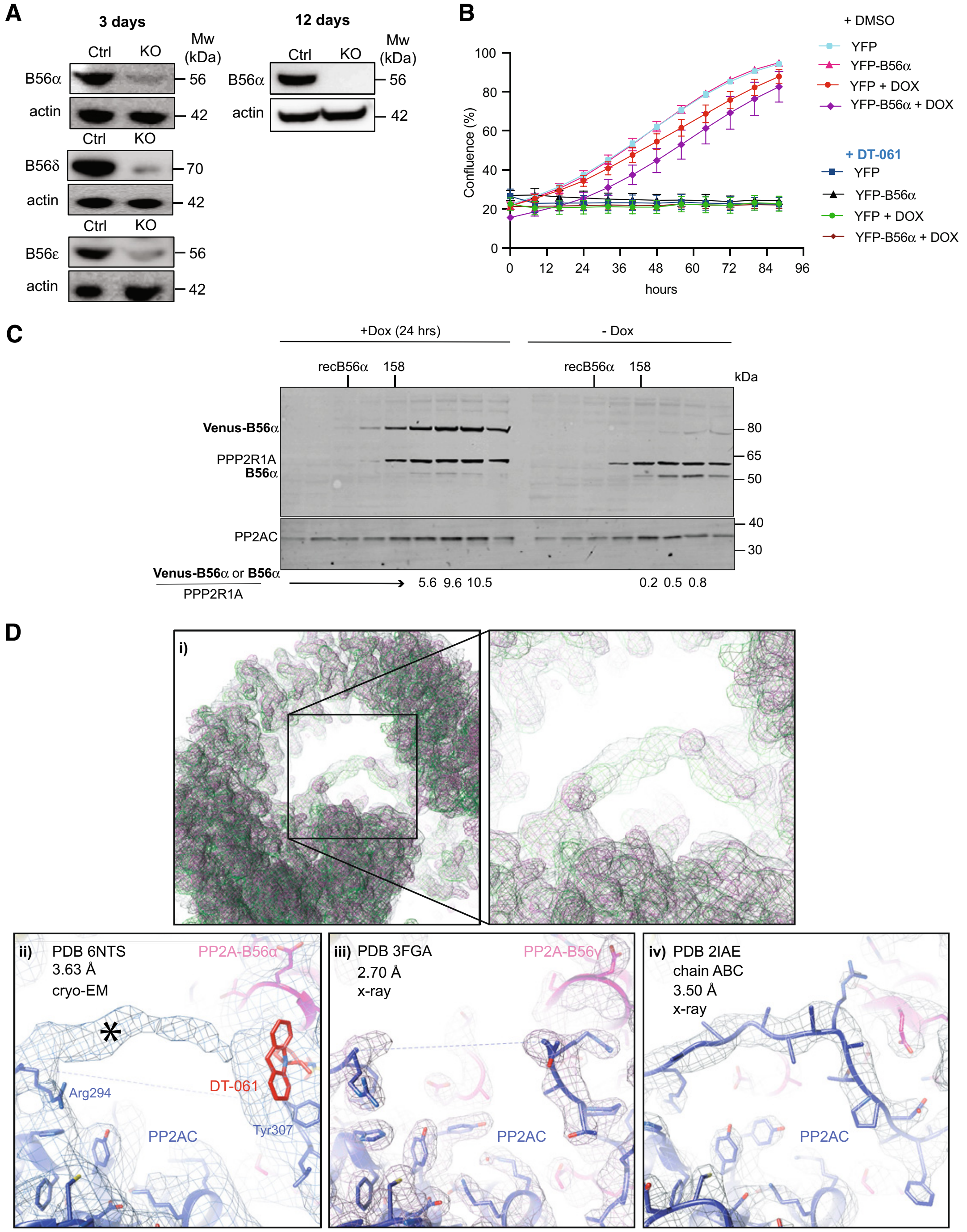
A
3 days
Ctrl KO
Mw (kDa)
B56α
56
actin
42
B56δ
70
B56ε
12 days
B
+ DMSO
YFP
YFP-B56α
YFP + DOX
YFP-B56α + DOX
+ DT-061
Confluence (%)
hours
C
+Dox (24 hrs)
- Dox
recB56α
158
kDa
Venus-B56α
PPP2R1A
PP2AC
Venus-B56α or B56α / PPP2R1A
5.6 9.6 10.5
0.2 0.5 0.8
D
i)
ii) PDB 6NTS 3.63 Å cryo-EM
PP2A-B56α
DT-061
Arg294
Tyr307
PP2AC
iii) PDB 3FGA 2.70 Å x-ray
PP2A-B56γ
iv) PDB 2IAE chain ABC 3.50 Å x-ray

Figure EV2.

Figure EV3. Compound titration and validation of CRISPR screen results.

A Viability of RPE1-hTERT P53$^{-/-}$ FLAG Cas9 cells with increasing concentrations of iHAP1 or DT-061 to determine LD20. Mean and SD of three biological replicates is shown.

B Protein depletion efficiency after RNAi-treatment. Actin was used as loading control. The experiment was performed three times.

C Sensitivity of HeLa Rod1 KO cells to 0.5 μM iHAP1. Western blot to confirm Rod1 depletion levels (left) and growth curves for the indicated conditions (right).

D Sensitivity of HeLa Rod1 KO to DT-061 or iHAP1 determined using SRB assay.

Data information: (C and D) Mean and SD of three independent experiments are shown.

 © 2022 The Authors

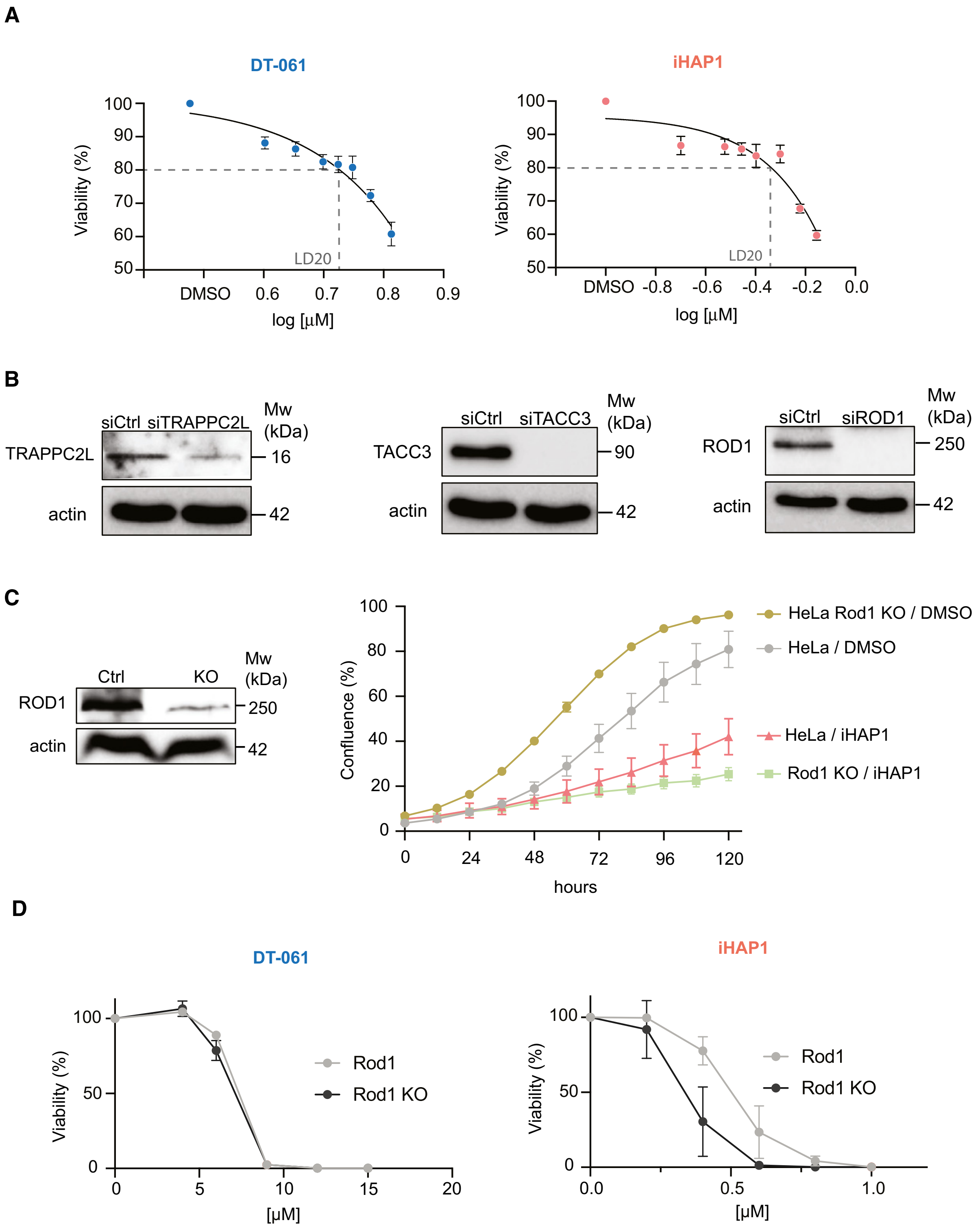
A
DT-061
Viability (%)
LD20
DMSO 0.6 0.7 0.8 0.9
log [µM]
iHAP1
DMSO -0.8 -0.6 -0.4 -0.2 0.0
B
siCtrl siTRAPPC2L
Mw (kDa)
TRAPPC2L
16
actin
42
siCtrl siTACC3
TACC3
90
siCtrl siROD1
ROD1
250
C
Ctrl KO
Confluence (%)
HeLa Rod1 KO / DMSO
HeLa / DMSO
HeLa / iHAP1
Rod1 KO / iHAP1
hours
D
Rod1
Rod1 KO
[µM]

Figure EV3.

Figure EV4. Analysis of mitotic phenotypes in cells treated with DT-061 or iHAP1.

A Representative spinning-disk confocal microscopy time-series of mitosis in U2OS cells stably expressing H2B-GFP/mCherry-α-tubulin following indicated treatments. Scale bar indicates 10 μm.

B Representative images of U2OS mitotic spindles in metaphase following indicated treatments, immunostained with α-tubulin antibody. DNA was counterstained with DAPI (cyan). Scale bar indicates 10 μm.

C Illustration describing the method used for measuring the amount of total, spindle and astral microtubules.

D Quantification of the number of microtubules in metaphase-arrested mitotic cells following indicated treatments. The mean and SD are plotted from three independent experiments (Total no. of cells = 48 (DMSO), 45 (30 μM DT-061+APC/C in.) and 48 (2 μM iHAP1+APC/C in.)). *P*-values were calculated using Student's *t*-test or Mann–Whitney *U* test (unpaired, two-tailed). See Materials and Methods for more details. ns, not significant, ****$P < 0.0001$.

© 2022 The Authors

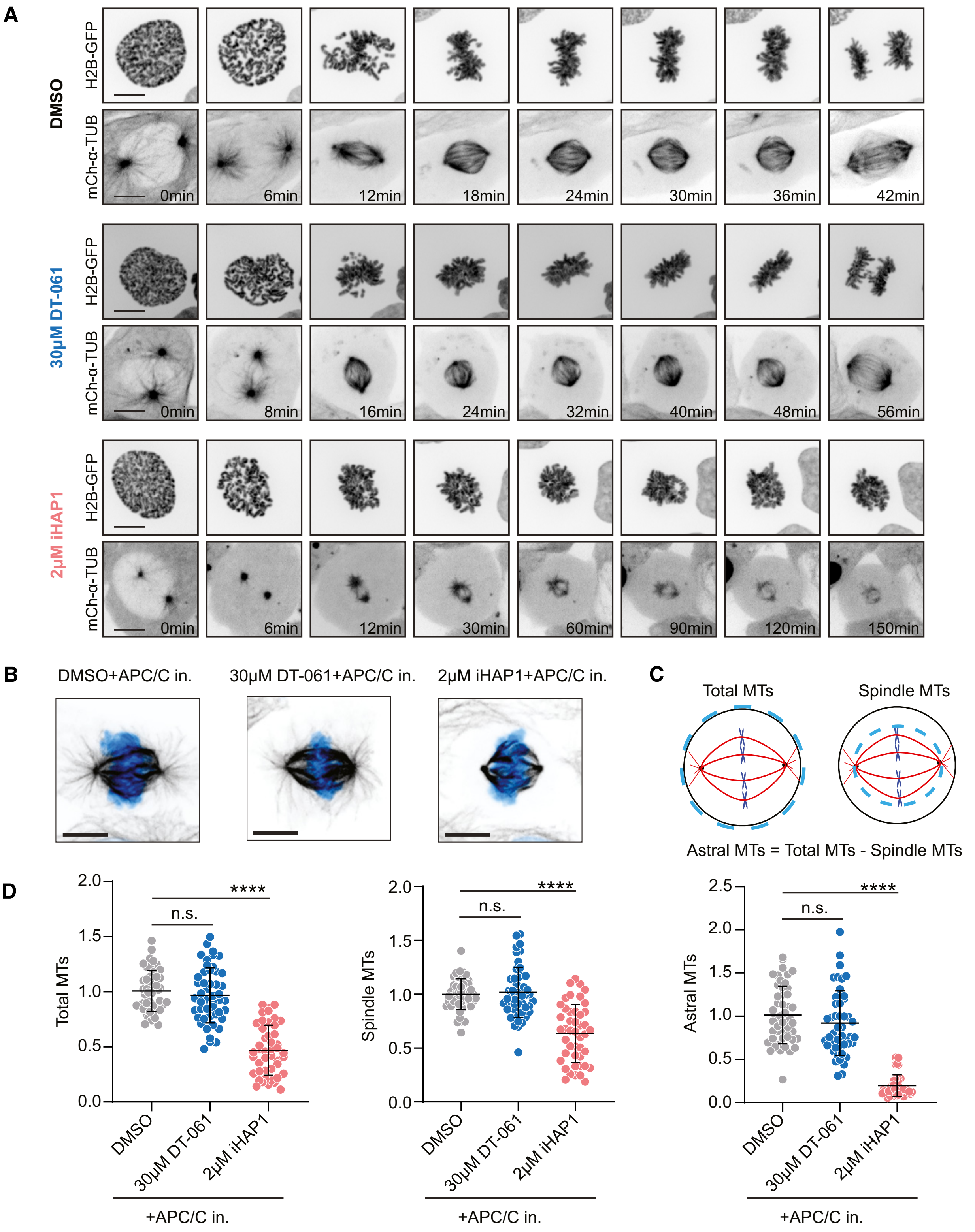
A
DMSO
H2B-GFP
mCh-α-TUB
0min
6min
12min
18min
24min
30min
36min
42min
30μM DT-061
8min
16min
32min
40min
48min
56min
2μM iHAP1
60min
90min
120min
150min
B
DMSO+APC/C in.
30μM DT-061+APC/C in.
2μM iHAP1+APC/C in.
C
Total MTs
Spindle MTs
Astral MTs = Total MTs - Spindle MTs
D
Total MTs
Spindle MTs
Astral MTs
n.s.
****
DMSO
30μM DT-061
2μM iHAP1
+APC/C in.

Figure EV4.

Figure EV5. Analysis of DT-061 effects on Golgi.

A Immunofluorescence analysis of Golgi using an antibody against GM130. The indicated cell lines were treated with 20 μM DT-061 for 30 min before fixation. Representative images shown. Scale bar 5 μm.

B Percentage of cells showing each phenotype after treatment with DT-061. Number of cells analyzed per condition from a single experiment is shown.

C Molar quantities of sphingolipid classes detected in MCF7 cells after feeding with the precursor sphingosine or treatment with vehicle (DMSO). MCF7 cells were co-treated with 15 μM DT-061, 1.5 μM iHAP1, 3 μg/ml Brefeldin A or vehicle (DMSO). The determined molar quantities of sphingolipid classes were normalized to that of PC. The presented values represent averages of five independent experiments, and statistical analysis was performed using an independent *t*-test. *$P < 0.05$, **$P < 0.01$, ***$P < 0.001$, ****$P < 0.0001$. Cer: ceramide, HexCer: hexosylceramide, PC: phosphatidylcholine and SM: sphingomyelin.

D Growth curves of HeLa cells treated with the indicated combinations and concentration of drugs. The results shown are representative of four independent experiments.

 © 2022 The Authors

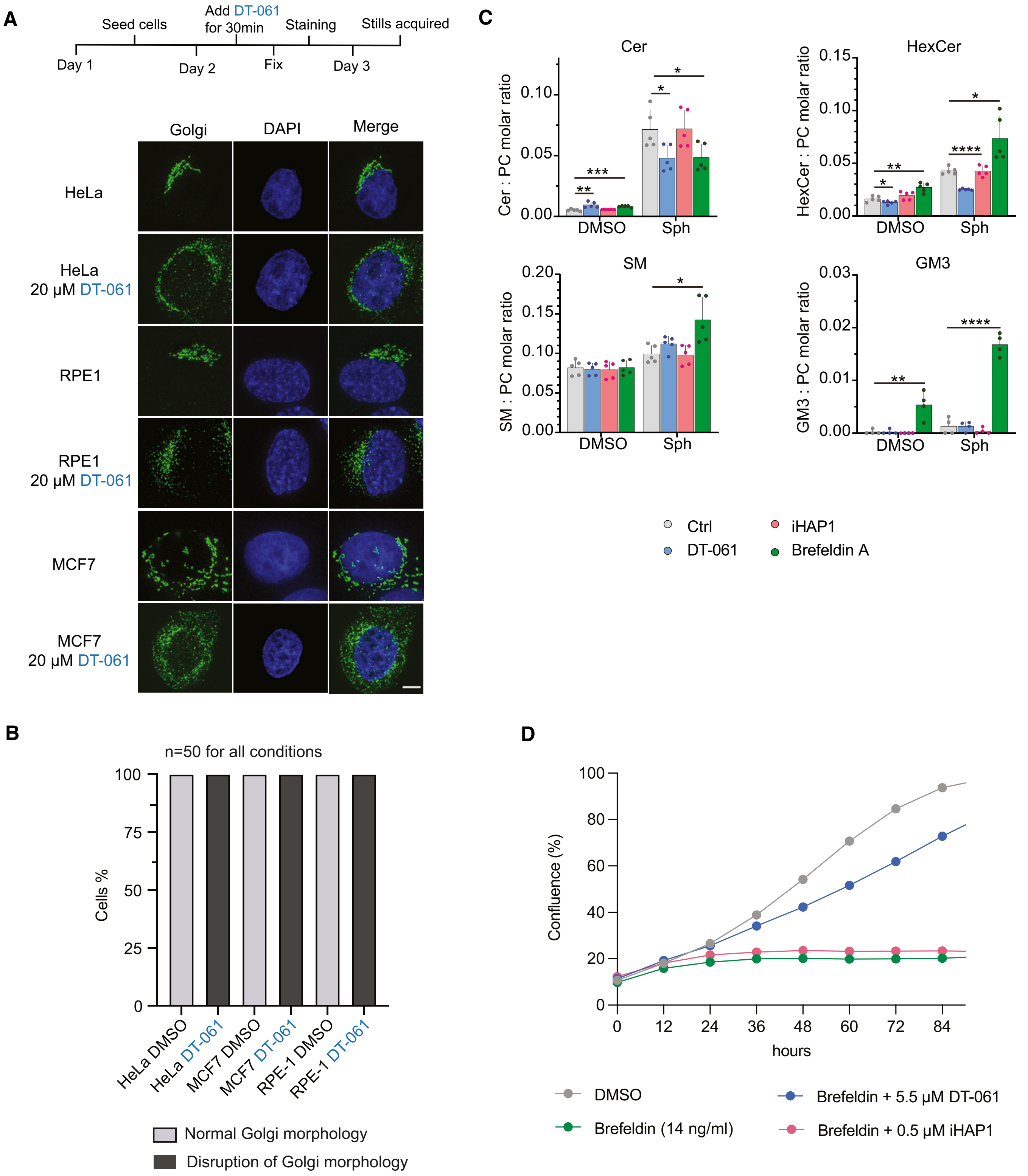
A
Seed cells
Add DT-061 for 30min
Staining
Stills acquired
Day 1
Day 2
Fix
Day 3
Golgi
DAPI
Merge
HeLa
HeLa 20 μM DT-061
RPE1
RPE1 20 μM DT-061
MCF7
MCF7 20 μM DT-061
B
n=50 for all conditions
Cells %
HeLa DMSO
HeLa DT-061
MCF7 DMSO
MCF7 DT-061
RPE-1 DMSO
RPE-1 DT-061
Normal Golgi morphology
Disruption of Golgi morphology
C
Cer
Cer : PC molar ratio
HexCer
HexCer : PC molar ratio
SM
SM : PC molar ratio
GM3
GM3 : PC molar ratio
DMSO
Sph
Ctrl
DT-061
iHAP1
Brefeldin A
D
Confluence (%)
hours
DMSO
Brefeldin (14 ng/ml)
Brefeldin + 5.5 μM DT-061
Brefeldin + 0.5 μM iHAP1

Figure EV5.

Funding Information – Author Query

AUTHOR: Please check that the funding details given below match the details given in the Acknowledgement and provide any missing funder details. In compliance with certain funding agencies (e.g., US Department of Energy), please add the FundRef DOI(s), which can be found, for example, at: http://www.wiley-vch.de/fundref/

1) Please check that the funding details given below list all funding agencies and grant numbers given in the Acknowledgement and provide any missing funders and funder DOIs where required by you funding agency with the proof corrections.
2) Please check that the funder agency name and DOI is provided according to the official FundRef listing.

FundRef funding agency name	FundRef Doi	Grant number
Novo Nordisk Fonden (NNF)	10.13039/501100009708	NNF14CC0001, NNF20OC0065098, NNF18OC0053124, NNF18CC0033876, NNF19OC0058504, NNF18OC0032996, NNF17CC0027852
Danish Cancer Society	10.13039/100015459	R146-A9322, R269-A15586-B17
Independent Research Fund Denmark	10.13039/501100011958	8021-00101B, 0134-00199B, 7016-00055, 6108-00542B
Swiss National Fund		P2EZP3_178624
Lundbeckfonden (Lundbeck Foundation)	10.13039/501100003554	2017-3212, R215-2015-4081
NIH/NIGMS		R35GM119455
NIH/NCI		R33CA225458
Wellcome Trust (WT)	10.13039/100010269	202811

© 2022 The Authors
