# Supplementary material for: Chemogenetic profiling reveals PP2A‐independent cytotoxicity of proposed PP2A activators iHAP1 and DT‐061
Source: EMBO J. 2022 Jun 13;41(14):e110611. doi: 10.15252/embj.2022110611 (PMC9289710; doi:10.15252/embj.2022110611)
Supplement: Supplementary file 6 — Movie EV1 [file EMBJ-41-e110611-s005.zip › Legend movie EV1.docx]

**Movie EV1**: Microtubule dynamics visualized in U2OS EB1-GFP cells following indicated treatments.
