# Supplementary material for: Chemogenetic profiling reveals PP2A‐independent cytotoxicity of proposed PP2A activators iHAP1 and DT‐061
Source: EMBO J. 2022 Jun 13;41(14):e110611. doi: 10.15252/embj.2022110611 (PMC9289710; doi:10.15252/embj.2022110611)
Supplement: Supplementary file 9 — Movie EV4 [file EMBJ-41-e110611-s002.zip › Legend movie EV4.docx]

**Movie EV4**: Mitosis in U2OS cells stably expressing H2B-GFP/mCherry-α-tubulin treated with 2µM iHAP1 for 20min before filming. DNA labelled by H2B-GFP on the left panel and microtubules labelled by mCherry-α-tubulin on the right panel.
