# Supplementary material for: Chemogenetic profiling reveals PP2A‐independent cytotoxicity of proposed PP2A activators iHAP1 and DT‐061
Source: EMBO J. 2022 Jun 13;41(14):e110611. doi: 10.15252/embj.2022110611 (PMC9289710; doi:10.15252/embj.2022110611)
Supplement: Supplementary file 10 — Movie EV5 [file EMBJ-41-e110611-s015.zip › Legend movie EV5.docx]

**Movie EV5**: Live cell imaging of HeLa cell expressing the Golgi marker treated with DT-061 (10 minutes time-lapse).
