# Supplementary material for: Chemogenetic profiling reveals PP2A‐independent cytotoxicity of proposed PP2A activators iHAP1 and DT‐061
Source: EMBO J. 2022 Jun 13;41(14):e110611. doi: 10.15252/embj.2022110611 (PMC9289710; doi:10.15252/embj.2022110611)
Supplement: Supplementary file 12 — Movie EV7 [file EMBJ-41-e110611-s021.zip › Legend movie EV7.docx]

**Movie EV7**: Live cell imaging of H358 cell expressing the Golgi marker treated with DT-061 (10 minutes time-lapse).
