# Supplementary material for: Chemogenetic profiling reveals PP2A‐independent cytotoxicity of proposed PP2A activators iHAP1 and DT‐061
Source: EMBO J. 2022 Jun 13;41(14):e110611. doi: 10.15252/embj.2022110611 (PMC9289710; doi:10.15252/embj.2022110611)

Source data

Chemogenetic profiling reveals PP2A-independent cytotoxicity of the PP2A activators iHAP1 and DT-061

Fig. 2A: original Western blot digital files.

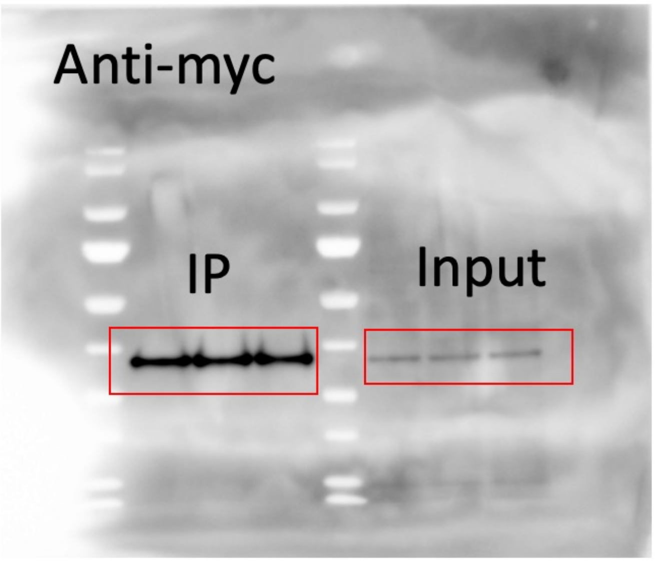

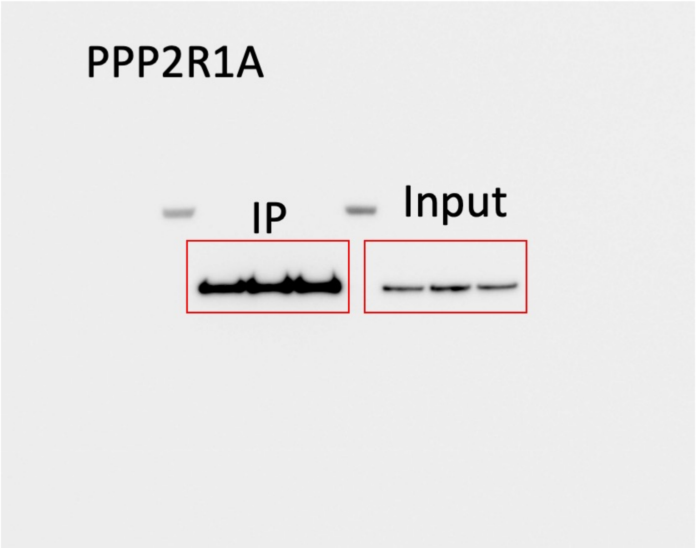

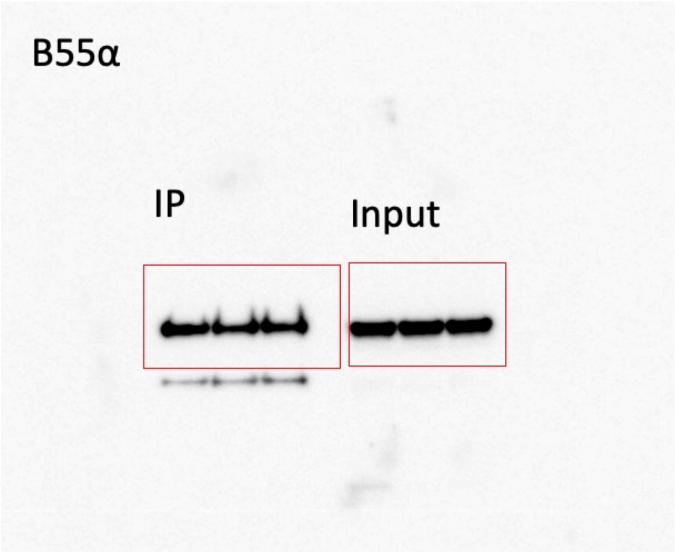

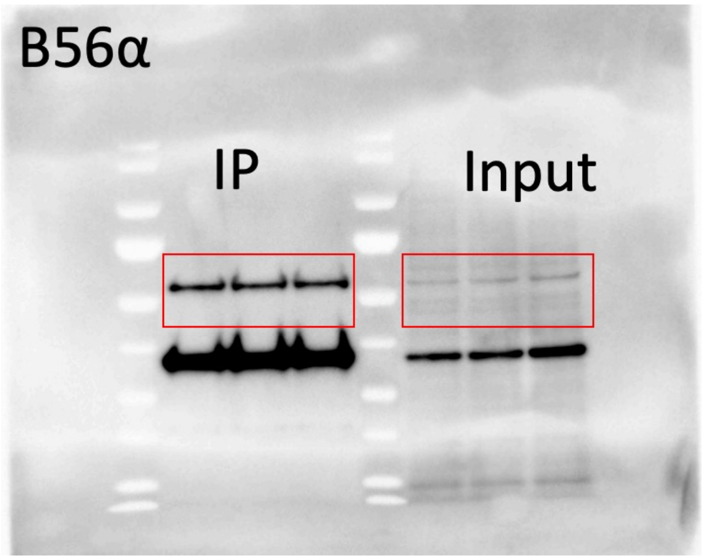

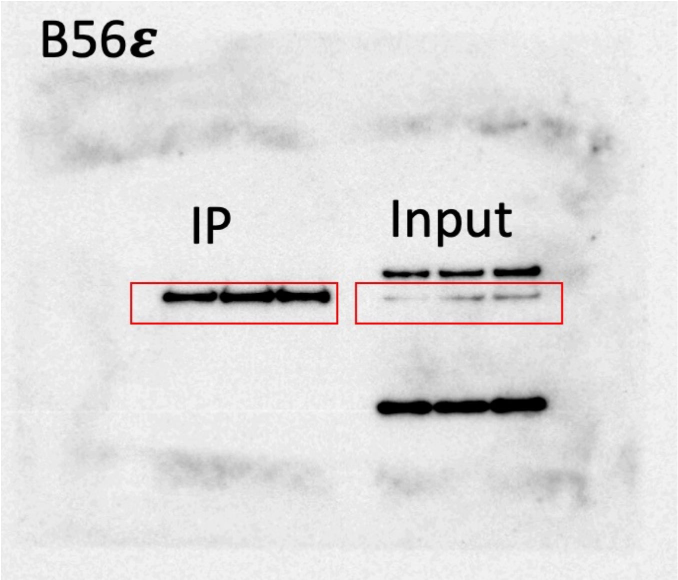

Fig. 2C: original Western blot digital files.

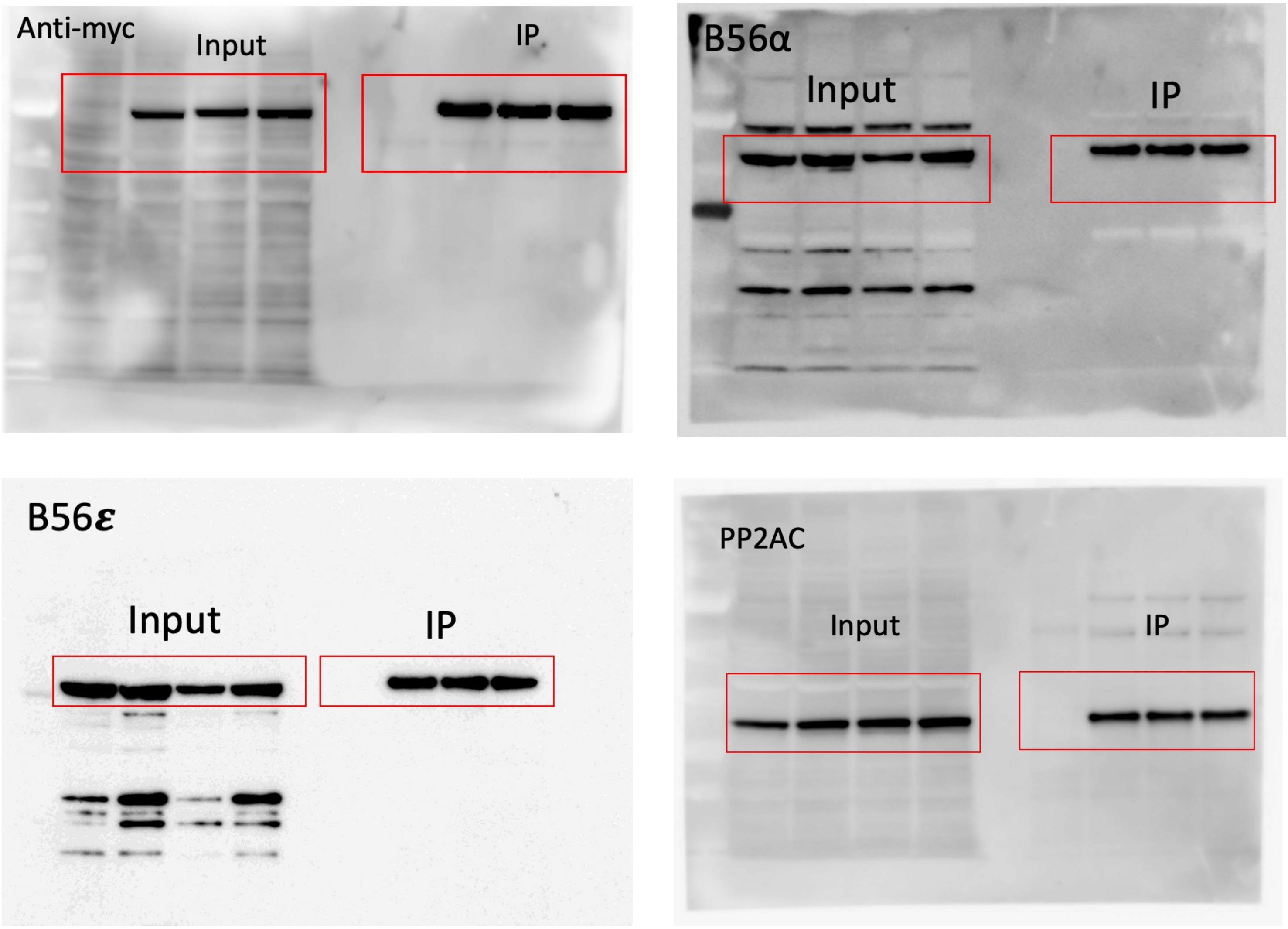

Supplement: Supplementary file 14 — Source Data for Figure 2 [file EMBJ-41-e110611-s006.zip › Source data.pdf]
