# Supplementary material for: Chemogenetic profiling reveals PP2A‐independent cytotoxicity of proposed PP2A activators iHAP1 and DT‐061
Source: EMBO J. 2022 Jun 13;41(14):e110611. doi: 10.15252/embj.2022110611 (PMC9289710; doi:10.15252/embj.2022110611)
Supplement: Supplementary file 21 [file EMBJ-41-e110611-s011.pdf]

THE EMBO JOURNAL

# Chemogenetic profiling reveals PP2A-independent cytotoxicity of the proposed PP2A activators iHAP1 and DT-061

Gianmatteo Vit, Joana Duro, Girish Rajendraprasad, Emil Hertz, Lay Katrine Holland, Melanie Weisser, Brennan McEwan, Blanca Lopez-Mendez, Paula Sotelo-Parilla, A. Arockia Jeyaprakash, Guillermo Montoya, Niels Mailand, Kenji Maeda, Arminja Kettenbach, Marin Barisic, and Jakob Nilsson
**DOI: 10.15252/embj.2022110611**

*Corresponding author(s): Jakob Nilsson (jakob.nilsson@cpr.ku.dk)*



## Review Timeline:

| | |
|---|---|
| Submission Date: | 7th Jan 22 |
| Editorial Decision: | 10th Feb 22 |
| Revision Received: | 6th May 22 |
| Editorial Decision: | 16th May 22 |
| Revision Received: | 18th May 22 |
| Accepted: | 19th May 22 |

*Editor: Hartmut Vodermaier*


## Transaction Report

Please note that the manuscript was previously reviewed at another journal and the reports were taken into account in the decision making process at The EMBO Journal. Since the original reviews are not subject to EMBO's transparent review process policy, the reports and author response cannot be published

**1st Editorial Decision** **10th Feb 2022**

Thank you again for transferring your manuscript together with referee reports from a another journal to The EMBO Journal. Following our own assessment of the study and your responses to the previous reviews, as well as discussions with expert editorial advisors, we conclude that a study revised along the lines suggested in your tentative response letter would be of interest to our readership. I am therefore inviting you to resubmit a new version incorporating the already obtained data and planned experiments, as well the various other clarifications. In particular, it should be good to include results obtained with the mentioned new antibody for holoenzyme immunoprecipitation. In addition, please rewrite particularly the abstract and extend the background introduction as appropriate for a stand-alone publication. Please do not hesitate to contact me in order to discuss any specific points ahead of resubmission.

Detailed information on preparing, formatting and uploading a revised manuscript can be found below and in our Guide to Authors - adhering to these guidelines as closely as possible should greatly facilitate editorial processing at the resubmission stage.

**Major new experimental data added to the revised manuscript**.

We thank the reviewers for the comments provided to improve our manuscript. We have added the following major new data to our manuscript to strengthen our arguments and address concerns raised by the reviewers:

1) We have analysed if perphenazine (PPZ) interacts with PPP2R1A as originally claimed in Gutierrez et al., 2014. This is important to establish, as PPZ is the parent compound providing the starting point for iHAP1 development and mode of action of DT-061. The fact that PPZ can directly bind to PPP2R1A was newer shown by Gutierrez et al. (the authors just referred to a personal communication). Using ITC and NMR, we could not detect any binding of PPZ to PPP2R1A and we see no effect of PPZ in enzymatic assays (new data in Fig. 1, EV1 and Appendix S1-3). Our data thus question the mode of action of PPZ as claimed by Gutierrez et al. This is in line with our inability to detect direct effects of iHAP1 and DT-061 on PP2A complexes.
2) We have analysed the effect of DT-061 on stabilizing reconstituted PP2A-B56α using mass photometry (MP) measurements. Using the same concentrations of holoenzyme as in Leonard et al., we see no effect of DT-061 (new data in Fig. 1D).
3) We analysed the effect of DT-061 on the endogenous holoenzymes in HeLa and H358 cell lines using size exclusion chromatography of cell extracts, since we could not identify an antibody able to immunoprecipitate endogenous PPP2R1A. Using this approach, we see a clear co-migration of endogenous B56α with PPP2R1A and no sign of free B56α, which would argue that there is no free B56α which DT-061 can act on. Furthermore, addition of DT-061 to cells and cell extract did not result in an increase in B56α co-migrating with PPP2R1A (new data in Appendix Fig. S6-7).
4) We have used an affinity tagged form of PPP2R1A to take a similar approach as in Morita et al. and Leonard et al. However, we did not detect an effect of PP2A-B56 composition by addition of DT-061 or iHAP1 (new data in Fig. 2C).
5) In an attempt to “mimic” the proposed mechanism of action of DT-061, we have used a stable cell line expressing inducible YFP-B56α. We have previously shown that this YFP-B56α is functional, as it can suppress RNAi depletion of all B56 isoforms. After inducing YFP-B56α expression, we see a large increase of PP2A-B56α holoenzyme formation by size-exclusion chromatography, yet no effect on cell growth. Under similar conditions DT-061 blocked cell growth (new data Fig. EV2B-C)
6) We have expanded our analysis of the cryo-EM structure reported in Leonard et al. and compared the tail of PP2AC to that of previous structures. All previous structures support the conclusion that the assignment of DT-061 is unambigious and can also be attributed to residues from the PP2AC tail (new analysis in Fig. EV2 and Appendix Fig. S8).
7) We show that the effect of iHAP1 on microtubules is not prevented by okadaic acid (we use it at concentrations that fully inactivate all PP2A complexes). This argues that the effect is not mediated by PP2A complexes (new data in Fig. 4)
8) We have expanded our analysis of the effect of DT-061 on ER and Golgi markers to H358 cells and have quantified all experiments. We have furthermore included okadaic acid treatments to determine if the effects we see with DT-061 are dependent on PP2A activity. The overall conclusion is that DT-061 affects Golgi and ER markers in both cell lines and largely independently of PP2A activity (new data in Fig. 6-7).

## 1st Revision - Editorial Decision 16th May 2022

Thank you for submitting your revised manuscript to The EMBO Journal. I have now carefully gone through your responses to the transferred original referee reports, and the changes made in response to my original decision letter. I am happy to say that we can now offer publication in our journal, as soon as a few remaining editorial points listed below have been addressed:

## 2nd Authors' Response to Reviewers 18th May 2022

The authors have made all requested editorial changes.

**Accepted** **19th May 2022**

Thank you for submitting your final revised manuscript for our consideration. I am pleased to inform you that we have now accepted it for publication in The EMBO Journal.

## EMBO Press Author Checklist

| |
|---|
| Corresponding Author Name: Jakob Nilsson |
| The EMBO Journal |
| Manuscript Number: EMBOJ-2022-110611 |

**USEFUL LINKS FOR COMPLETING THIS FORM**
The EMBO Journal - Author Guidelines
EMBO Reports - Author Guidelines
Molecular Systems Biology - Author Guidelines
EMBO Molecular Medicine - Author Guidelines

**Reporting Checklist for Life Science Articles (updated January 2022)**
This checklist is adapted from Materials Design Analysis Reporting (MDAR) Checklist for Authors. MDAR establishes a minimum set of requirements in transparent reporting in the life sciences (see Statement of Task: 10.31222/osf.io/9sm4x). Please follow the journal's guidelines in preparing your manuscript.
**Please note that a copy of this checklist will be published alongside your article.**

**Abridged guidelines for figures**
**1. Data**
The data shown in figures should satisfy the following conditions:
- the data were obtained and processed according to the field's best practice and are presented to reflect the results of the experiments in an accurate and unbiased manner.
- ideally, figure panels should include only measurements that are directly comparable to each other and obtained with the same assay.
- plots include clearly labeled error bars for independent experiments and sample sizes. Unless justified, error bars should not be shown for technical replicates.
- if n<5, the individual data points from each experiment should be plotted. Any statistical test employed should be justified.
- Source Data should be included to report the data underlying figures according to the guidelines set out in the authorship guidelines on Data Presentation.

**2. Captions**
Each figure caption should contain the following information, for each panel where they are relevant:
- a specification of the experimental system investigated (eg cell line, species name).
- the assay(s) and method(s) used to carry out the reported observations and measurements.
- an explicit mention of the biological and chemical entity(ies) that are being measured.
- an explicit mention of the biological and chemical entity(ies) that are altered/varied/perturbed in a controlled manner.
- the exact sample size (n) for each experimental group/condition, given as a number, not a range;
- a description of the sample collection allowing the reader to understand whether the samples represent technical or biological replicates (including how many animals, litters, cultures, etc.).
- a statement of how many times the experiment shown was independently replicated in the laboratory.
- definitions of statistical methods and measures:
  - common tests, such as t-test (please specify whether paired vs. unpaired), simple χ2 tests, Wilcoxon and Mann-Whitney tests, can be unambiguously identified by name only, but more complex techniques should be described in the methods section;
  - are tests one-sided or two-sided?
  - are there adjustments for multiple comparisons?
  - exact statistical test results, e.g., P values = x but not P values < x;
  - definition of 'center values' as median or average;
  - definition of error bars as s.d. or s.e.m.

**Please complete ALL of the questions below.**
**Select "Not Applicable" only when the requested information is not relevant for your study.**

### Materials

| **Newly Created Materials** | **Information included in the manuscript?** | **In which section is the information available?** (Reagents and Tools Table, Materials and Methods, Figures, Data Availability Section) |
|---|---|---|
| New materials and reagents need to be available; do any restrictions apply? | Yes | We have generated a number of stable cell lines for this study that can be requested. We have indicated this in the acknowledgment section. |

| **Antibodies** | **Information included in the manuscript?** | **In which section is the information available?** (Reagents and Tools Table, Materials and Methods, Figures, Data Availability Section) |
|---|---|---|
| For **antibodies** provide the following information:<br>- Commercial antibodies: RRID (if possible) or supplier name, catalogue number and or/clone number<br>- Non-commercial: RRID or citation | Yes | We have provided supplier name and catalogue number for all antibodies used in this study. A separate section in materials and methods has this information collected. |

| **DNA and RNA sequences** | **Information included in the manuscript?** | **In which section is the information available?** (Reagents and Tools Table, Materials and Methods, Figures, Data Availability Section) |
|---|---|---|
| **Short novel DNA or RNA including primers, probes**: provide the sequences. | Not Applicable | We have not used DNA or RNA primers. Constructs used are from previous papers or generated through gateway cloning for the affinity tagged PPP2R1A. |

| **Cell materials** | **Information included in the manuscript?** | **In which section is the information available?** (Reagents and Tools Table, Materials and Methods, Figures, Data Availability Section) |
|---|---|---|
| **Cell lines:** Provide species information, strain. Provide accession number in repository **OR** supplier name, catalog number, clone number, and/**OR** RRID. | Yes | We have provided ATCC ID for HeLa, U2OS and H358 cells used in this study. For other cell lines we have provided references. This information is available in the method section under Cell Culture methods |
| **Primary cultures:** Provide species, strain, sex of origin, genetic modification status. | Not Applicable | |
| Report if the cell lines were recently **authenticated** (e.g., by STR profiling) and tested for mycoplasma contamination. | Yes | We have not recently authenticated cells or tested for mycoplasma. The H358 was bought from ATCC during revision. |

| **Experimental animals** | **Information included in the manuscript?** | **In which section is the information available?** (Reagents and Tools Table, Materials and Methods, Figures, Data Availability Section) |
|---|---|---|
| **Laboratory animals or Model organisms:** Provide species, strain, sex, age, genetic modification status. Provide accession number in repository **OR** supplier name, catalog number, clone number, **OR** RRID. | Not Applicable | |
| **Animal observed in or captured from the field:** Provide species, sex, and age where possible. | Not Applicable | |
| Please detail **housing and husbandry conditions**. | Not Applicable | |

| **Plants and microbes** | **Information included in the manuscript?** | **In which section is the information available?** (Reagents and Tools Table, Materials and Methods, Figures, Data Availability Section) |
|---|---|---|
| **Plants:** provide species and strain, ecotype and cultivar where relevant, unique accession number if available, and source (including location for collected wild specimens). | Not Applicable | |
| **Microbes:** provide species and strain, unique accession number if available, and source. | Not Applicable | |

| **Human research participants** | **Information included in the manuscript?** | **In which section is the information available?** (Reagents and Tools Table, Materials and Methods, Figures, Data Availability Section) |
|---|---|---|
| If collected and within the bounds of privacy constraints report on age, sex and gender or ethnicity for all study participants. | Not Applicable | |

| **Core facilities** | **Information included in the manuscript?** | **In which section is the information available?** (Reagents and Tools Table, Materials and Methods, Figures, Data Availability Section) |
|---|---|---|
| If your work benefited from core facilities, was their service mentioned in the acknowledgments section? | Yes | Acknowledgements. |

### Design

| **Study protocol** | **Information included in the manuscript?** | **In which section is the information available?** (Reagents and Tools Table, Materials and Methods, Figures, Data Availability Section) |
|---|---|---|
| If study protocol has been **pre-registered, provide DOI in the manuscript**. For clinical trials, provide the trial registration number **OR** cite DOI. | Not Applicable | |
| Report the **clinical trial registration number** (at ClinicalTrials.gov or equivalent), where applicable. | Not Applicable | |

| **Laboratory protocol** | **Information included in the manuscript?** | **In which section is the information available?** (Reagents and Tools Table, Materials and Methods, Figures, Data Availability Section) |
|---|---|---|
| Provide DOI OR other citation details if **external detailed step-by-step protocols** are available. | Not Applicable | |

| **Experimental study design and statistics** | **Information included in the manuscript?** | **In which section is the information available?** (Reagents and Tools Table, Materials and Methods, Figures, Data Availability Section) |
|---|---|---|

| | | |
|---|---|---|
| Include a statement about **sample size** estimate even if no statistical methods were used. | Yes | No statistical method was used to predetermine sample size. |
| Were any steps taken to minimize the effects of subjective bias when allocating animals/samples to treatment (e.g. **randomization procedure**)? If yes, have they been described? | Yes | We have not used randomisation procedures. Samples were analysed by the scientist performning the experiment. |
| Include a statement about **blinding** even if no blinding was done. | Yes | We have included a statement that no blinding was performed in the methods section. |
| Describe **inclusion/exclusion criteria** if samples or animals were excluded from the analysis. Were the criteria pre-established?<br><br>If sample or data points were omitted from analysis, report if this was due to attrition or intentional exclusion and provide justification. | Yes | We have only excluded experiments were the controls did not work. |
| For every figure, are **statistical tests** justified as appropriate? Do the data meet the assumptions of the tests (e.g., normal distribution)? Describe any methods used to assess it. Is there an estimate of variation within each group of data? Is the variance similar between the groups that are being statistically compared? | Yes | We have used statistical tests in Fig. 2B-D (mass spectrometry data), Fig. 4C and E (microtubule dynamics), Fig. 8B and EV5C (lipodomics). To the best of our knowledge the correct statistical tests have been used. Test for normality and statistical methods described in methods section. |

| Sample definition and in-laboratory replication | Information included in the manuscript? | In which section is the information available?<br>(Reagents and Tools Table, Materials and Methods, Figures, Data Availability Section) |
|---|---|---|
| In the figure legends: state number of times the experiment was **replicated** in laboratory. | Yes | We have provided this information in the figure legend |
| In the figure legends: define whether data describe **technical or biological replicates**. | Yes | We have provided this information in the figure legend |

## Ethics

| Ethics | Information included in the manuscript? | In which section is the information available?<br>(Reagents and Tools Table, Materials and Methods, Figures, Data Availability Section) |
|---|---|---|
| Studies involving **human participants**: State details of **authority granting ethics approval** (IRB or equivalent committee(s), provide reference number for approval. | Not Applicable | |
| Studies involving **human participants**: Include a statement confirming that **informed consent** was obtained from all subjects and that the experiments conformed to the principles set out in the WMA Declaration of Helsinki and the Department of Health and Human Services Belmont Report. | Not Applicable | |
| Studies involving **human participants:** For publication of **patient photos**, include a statement confirming that consent to publish was obtained. | Not Applicable | |
| Studies involving experimental **animals**: State details of **authority granting ethics approval** (IRB or equivalent committee(s), provide reference number for approval. Include a statement of compliance with ethical regulations. | Not Applicable | |
| Studies involving **specimen and field samples:** State if relevant **permits** obtained, provide details of authority approving study; if none were required, explain why. | Not Applicable | |

| Dual Use Research of Concern (DURC) | Information included in the manuscript? | In which section is the information available?<br>(Reagents and Tools Table, Materials and Methods, Figures, Data Availability Section) |
|---|---|---|
| Could your study fall under dual use research restrictions? Please check biosecurity documents and list of **select agents and toxins** (CDC): https://www.selectagents.gov/sat/list.htm | Not Applicable | |
| If you used a select agent, is the security level of the lab appropriate and reported in the manuscript? | Not Applicable | |
| If a study is subject to dual use research of concern regulations, is the name of the **authority granting approval and reference number** for the regulatory approval provided in the manuscript? | Not Applicable | |

## Reporting

The MDAR framework recommends adoption of discipline-specific guidelines, established and endorsed through community initiatives. Journals have their own policy about requiring specific guidelines and recommendations to complement MDAR.

| Adherence to community standards | Information included in the manuscript? | In which section is the information available?<br>(Reagents and Tools Table, Materials and Methods, Figures, Data Availability Section) |
|---|---|---|
| State if relevant guidelines or checklists (e.g., **ICMJE, MIBBI, ARRIVE, PRISMA**) have been followed or provided. | Not Applicable | |
| For **tumor marker prognostic studies,** we recommend that you follow the **REMARK** reporting guidelines (see link list at top right). See author guidelines, under 'Reporting Guidelines'. Please confirm you have followed these guidelines. | Not Applicable | |
| For **phase II and III randomized controlled trials**, please refer to the **CONSORT** flow diagram (see link list at top right) and submit the CONSORT checklist (see link list at top right) with your submission. See author guidelines, under 'Reporting Guidelines'. Please confirm you have submitted this list. | Not Applicable | |

## Data Availability

| Data availability | Information included in the manuscript? | In which section is the information available?<br>(Reagents and Tools Table, Materials and Methods, Figures, Data Availability Section) |
|---|---|---|
| Have **primary datasets** been deposited according to the journal's guidelines (see 'Data Deposition' section) and the respective accession numbers provided in the Data Availability Section? | Yes | Yes. A data availibity statement is provided at the end of methods |
| Were **human clinical and genomic datasets** deposited in a public access-controlled repository in accordance to ethical obligations to the patients and to the applicable consent agreement? | Not Applicable | |
| Are **computational models** that are central and integral to a study available without restrictions in a machine-readable form? Were the relevant accession numbers or links provided? | Not Applicable | |
| If publicly available data were reused, provide the respective **data citations in the reference list**. | Yes | Yes we have used the publicly available structure of PP2A-B56-DT-061 and acknowledged this through refence. |
